# A One Pot Synthesis of Novel Bioactive Tri-Substitute-Condensed-Imidazopyridines that Targets Snake Venom Phospholipase A_2_

**DOI:** 10.1371/journal.pone.0131896

**Published:** 2015-07-21

**Authors:** Nirvanappa C. Anilkumar, Mahalingam S. Sundaram, Chakrabhavi Dhananjaya Mohan, Shobith Rangappa, Krishna C. Bulusu, Julian E. Fuchs, Kesturu S. Girish, Andreas Bender, Basappa, Kanchugarakoppal S. Rangappa

**Affiliations:** 1 Laboratory of Chemical Biology, Department of Chemistry, Bangalore University, Central College campus, Palace Road, Bangalore-560 001, Karnataka, India; 2 Department of Studies in Biochemistry, University of Mysore, Mysore-570 006, Karnataka, India; 3 Department of Studies in Chemistry, University of Mysore, Mysore-570 006, Karnataka, India; 4 Frontier Research Center for Post-genome Science and Technology Hokkaido University, Sapporo, 060–0808, Japan; 5 Centre for Molecular Science Informatics, Department of Chemistry, University of Cambridge, Lensfield Road, CB2 1EW, Cambridge, United Kingdom; 6 Department of Studies and Research in Biochemistry, Tumkur University, Tumkur-572 103, Karnataka, India; Universidade Federal do Rio de Janeiro, BRAZIL

## Abstract

Drugs such as necopidem, saripidem, alpidem, zolpidem, and olprinone contain nitrogen-containing bicyclic, condensed-imidazo[1,2-α]pyridines as bioactive scaffolds. In this work, we report a high-yield one pot synthesis of 1-(2-methyl-8-aryl-substitued-imidazo[1,2-α]pyridin-3-yl)ethan-1-onefor the first-time. Subsequently, we performed *in silico* mode-of-action analysis and predicted that the synthesized imidazopyridines targets Phospholipase A_2_ (PLA_2_). *In vitro* analysis confirmed the predicted target PLA_2_ for the novel imidazopyridine derivative1-(2-Methyl-8-naphthalen-1-yl-imidazo [1,2-α]pyridine-3-yl)-ethanone (compound **3f**) showing significant inhibitory activity towards snake venom PLA_2_ with an IC_50_ value of 14.3 μM. Evidently, the molecular docking analysis suggested that imidazopyridine compound was able to bind to the active site of the PLA_2_ with strong affinity, whose affinity values are comparable to nimesulide. Furthermore, we estimated the potential for oral bioavailability by Lipinski's Rule of Five. Hence, it is concluded that the compound **3f** could be a lead molecule against snake venom PLA_2_.

## Introduction

**Fig 1:**
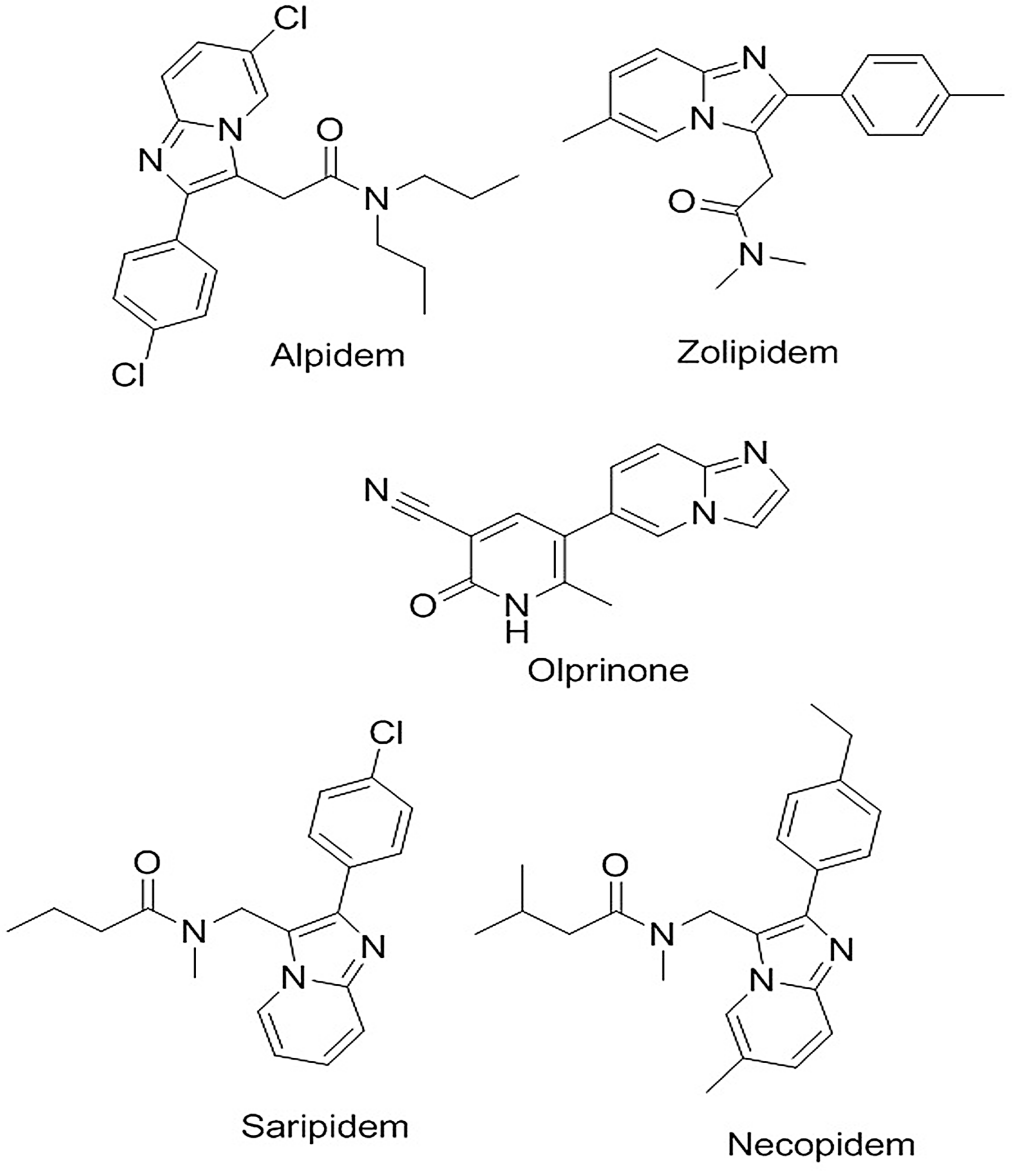
Structure of biologically active imidazo[1, 2-α]pyridines.

Imidazole derivatives are the distinct class of heterocyclic compounds which exhibit remarkable pharmacological activities across a wide range of therapeutic targets [1, 2]. Research in the previous decade demonstrated that bicyclic condensation of imidazo[1,2-a]pyridines possess multiple therapeutic properties including anti-cytomegalo-zoster, anti-microbial, anti-cancer, anti-inflammatory and anti-protozoal activities [3–7]. Imidazo[1,2-a]pyridine ring is a component of anxiolytic and sedative drugs such as necopidem, saripidem, alpidem, zolpidem, and olprinone (Fig 1) [8]. These reports suggest the critical role of imidazopyridines in medicinal chemistry and requirement of easy route for the synthesis of imidazopyridines with improved efficacy. Initially, copper catalyzed synthesis of imidazopyridines was reported by coupling of 2-aminopyridine with benzaldehyde and propiolic acid as a source of alkyne [9]. Similarly, NaAuCl_4_ and Cu(OTf)_2_ catalyzed synthesis of imidazo[1,2-α]pyridine were reported. The silver-catalyzed synthesis of substituted-3-methylimidazo[1,2-α]pyridines by cyclo-isomerization was reported at milder reaction conditions by using N-(prop-2-yn-1-yl)-pyridine-2-amines [10]. Recently, Dimauro et al reported the Pd(II) and copper iodide catalyzed synthesis of 2-benzylimidazo[1,2-a]pyridines using 2-amino-1-(2-propynyl)pyridinium bromide, aryl halides and triethylamine [11].

Additionally, the indium(III) bromide catalyzed multi-component one pot synthesis of imidazo[1,2-a]pyridines by means of 2-aminopyridine, aldehyde and alkyne was reported [6]. In an another study, Lamblin and colleagues reported theone pot, four-component, microwave assisted, MgCl_2_ catalyzed synthesis of imidazopyridines by Ugi-type cyclization of 2-aminopyridine boronic acid pinacol ester, aldehyde and isocyanide, followed by Suzuki coupling with different aryl halides [12].

In summary, most of the reported synthetic routes of imidazopyridines involve the use of a catalyst and an alkyne, or the eventual Suzuki-Miyaura cross-coupling reactions. In the present work, we developed a one pot two-step synthesis of tri-substituted-condensed-imidazopyridines for the first time without using a catalyst for the cyclization, followed by Suzuki coupling reaction. Further, *in silico* mode of action analysis predicted phospholipase A_2_ (PLA_2_) as a potential protein target of title compounds, which has subsequently been validated experimentally.

## Materials and Methods

### Chemicals/reagents

*Vipera russelli* (RV) venom was obtained from Hindustan snake park, Kolkata, India. Solvents and reagents used in this study were of analytical grade and were purchased from Sigma-Aldrich, St. Louis, USA. 1,2-bis(heptanoylthio)glycerophosphocholine was purchased from Santa Cruz Biotechnology, Inc. Texas, USA. The VRV-PLA_2_-VIII was isolated from RV according to the method of Kasturi and Gowda [13].

### General procedure for the synthesis of 1-[(6a-l)-2-methyl-imidazo[1, 2-α]pyridine-3-yl]ethanone derivatives

The mixture of 3-bromopyridine-2-amine (200 mg, 0.08mmol), 3-bromopentane-2, 4-dione (142 mg, 0.08 mmol) and 4 mL of tetrahydrofuran (THF) were taken in a sealed tube and heated at 60°C for 4 h and the reaction was monitored by TLC. After the completion of reaction, boronic acids (0.08 mmol) were added along with Pd(dppf)Cl_2_ (0.002 mmol) and K_2_CO_3_ (0.17 mmol). Finally, 1mL of water was added and the reaction was continued for 4 h at 60°C. Solvent was evaporated to obtain the crude product and further it was purified by passing through the column chromatography using hexane and ethyl acetate as solvents.

All IR spectra were obtained in KBr disc on a Shimadzu FT-IR 157 Spectrometer. ^1^H and ^13^C NMR spectra were recorded on a Bruker WH-200 (400MZ) spectrometer in CDCl_3_ or DMSO-d_6_ as solvent, using TMS as an internal standard and chemical shifts are expressed as ppm. Mass spectra were determined using LC-MS. (Shimadzu). The elemental analyses were carried out using an Elemental Vario Cube CHNS rapid Analyser. The progress of the reaction was monitored by TLC pre-coated silica gel G plates. Melting points were determined in a melting point apparatus and were uncorrected. The structures of novel imidazopyridine derivatives are presented in Table 1. Spectra (S1 Data) and characterization data is provided as supplementary data (S2 Data).

**Table 1:** Physical data of the tri-substituted-condensed-imidazopyridines and IC_50_ values towards the binding of PLA_2_.

Entry	Boronic acids	Products	Yield (%)	Mp (°C)	IC_50_ (μM)	
					RV venom	VRV-PLA2-VIII
3a	(4-chloro-3-(trifluoromethyl)phenyl)boronic acid	1-(8-(4-chloro-3-(trifluoromethyl)phenyl)-2-methylimidazo[1,2-a]pyridin-3-yl)ethanone	70	141–143	110	155
3b	(4-(benzyloxy)-3-fluorophenyl)boronic acid	1-(8-(4-(benzyloxy)-3-fluorophenyl)-2-methylimidazo[1,2-a]pyridin-3-yl)ethanone	78	154–156	NS	140
3c	Phenylboronic acid	1-(2-methyl-8-phenylimidazo[1,2-a]pyridin-3-yl)ethanone	75	160–162	194	246
3d	(3-chlorophenyl)boronic acid	1-(8-(3-chlorophenyl)-2-methylimidazo[1,2-a]pyridin-3-yl)ethanone	80	114–116	59.1	89.1
3e	(3-methoxyphenyl)boronic acid	1-(8-(3-methoxyphenyl)-2-methylimidazo[1,2-a]pyridin-3-yl)ethanone	81	116–119	46.4	65.3
3f	Naphthalen-1-ylboronic acid	1-(2-methyl-8-(naphthalen-1-yl)imidazo[1,2-a]pyridin-3-yl)ethanone	86	179–182	14.3	23.1
3g	(4-chlorophenyl)boronic acid	1-(8-(4-chlorophenyl)-2-methylimidazo[1,2-a]pyridin-3-yl)ethanone	82	111–114	219	194.8
3h	(3-(cyclopentylcarbamoyl)pentyl)boronic acid	4-(3-acetyl-2-methylimidazo[1,2-a]pyridin-8-yl)-N-cyclopentyl-2-ethylbutanamide	87	117–119	105	114.4
3i	(2-fluoro-3-methoxyphenyl)boronic acid	1-(8-(2-fluoro-3-methoxyphenyl)-2-methylimidazo[1,2-a]pyridin-3-yl)ethanone	86	190–192	123	134.9
3j	o-tolylboronic acid	1-(2-methyl-8-(o-tolyl)imidazo[1,2-a]pyridin-3-yl)ethanone	80	157–159	166	189
3k	(4-(trifluoromethyl)phenyl)boronic acid	1-(2-methyl-8-(4-(trifluoromethyl)phenyl)imidazo[1,2-a]pyridin-3-yl)ethanone	76	121–123	NS	NS
3l	(4-ethylphenyl)boronic acid	1-(8-(4-ethylphenyl)-2-methylimidazo[1,2-a]pyridin-3-yl)ethanone	79	169–172	NS	43.2

NS: Not Significant

### Cheminformatics based rationalization

Utilizing the increasing amount of available bioactivity data, we were able to rationalize the mode-of-action for the imidazopyridines using *in silico* approaches, which is currently of interest for chemogenomics studies [14]. To obtain the most probable target for imidazopyridines, we applied the Laplacian-modified Naïve Bayesian classifier and predicted the potential targets as developed by Koutsoukas et al [15, 16]. This classifier was trained on a large dataset extracted from ChEMBL, comprising approximately 190,000 bioactive compounds covering 477 human protein targets [17]. A score cut-off of 10 was applied to the predictions, meaning that predictions with a score of 0.2 or greater were considered to be possible protein targets of the compound.

### *In vitro* PLA_2_ inhibition assay

#### a) Indirect haemolytic activity

Indirect haemolytic activity was determined according to the method described by Boman and Kaletta [18]. Briefly, packed human erythrocytes were repeatedly washed with phosphate buffered saline (PBS, 10 mM pH 7.4) and the assay stock was prepared by mixing packed human erythrocytes, egg yolk and PBS (1:1:8; v/v/v). The stock suspension (200 μL) was incubated independently with 1 μg of RV venom in a total volume of 300 μL for 1 h at 37°C. The reaction was terminated by adding 1.7 mL of ice-cold PBS and centrifuged at 160 ×*g* for 10 min. The amount of haemoglobin released in the supernatant was measured at 540 nm. Stock suspension (200 μL) with 1.8 mL of ice-cold PBS alone was considered as 0% lysis. The activity was expressed as percent haemolysis against 100% lysis of cells by water. For inhibition studies, 1 μg of RV venom was pre-incubated with different concentrations of **3a-l** (0–500 μM) for 10 min at 37°C and necessary controls were maintained in the respective groups. Compounds were dissolved in DMSO and further diluted in PBS and final concentration of DMSO was less than 0.05% in the reaction mixture.

#### b) VRV-PLA_2_-VIII inhibition assay

The assay was carried out according to the method of Petrovic et al [19] using isolated PLA_2_ (VRV-PLA_2_-VIII) and 1,2-bis(heptanoylthio)glycerophosphocholineas substrate [18]. Briefly, VRV-PLA_2_-VIII (5 μg) was pre-incubated with different concentrations of **3a-l** (0–100 μM) for 10 min at 37°C in 96 well microtiter plate. Further, PLA_2_ substrate (2 mM) containing 1 mM DTNB was added to each well to a final reaction volume of 100 μL with assay buffer (50 mM Tris-HCl, pH 7.5 containing 150 mM KCl and 10 mM CaCl_2_) and incubated for 60 min at room temperature. The resulting absorbances were measured at 415 nm and 600 nm.

### Molecular docking studies

*In silico* molecular docking was performed based on the X-ray structure of Russell's viper PLA_2_ in complex with nimesulide with a resolution of 1.1Å (PDB: 1ZWP) [20, 21].The structures were prepared by removing the sulphate ions and bound methanol to subject it to docking in MOE [21].The synthesized molecules were docked to the active site of PLA_2_using the co-crystallized nimesulide as starting point. To enforce reasonable docking poses, we introduced a pharmacophore filter to discard poses not showing a hydrogen bond acceptor feature at the position of the nitro group of nimesulide. We applied the standard flexible docking work-flow implemented in MOE including placement with Triangle Matcher, primary scoring with London dG and subsequent refinement using the mmff94x forcefield [22]. The highest scoring pose for each compound according to GBVI/WSA dG was finally considered. Resulting poses were visualized in Pymol [23].

### Structure-activity relationships

We calculated molecular descriptors for all twelve newly synthesized compounds using MOE aiming to identify correlations between physico-chemical properties and biological activities. Molecular were treated in their predicted biologically active conformation from docking. Correlations were calculated as non-parametric Spearman rank correlation coefficient. Furthermore, we estimated the potential for oral bioavailability by Lipinski's Rule of Five. Descriptors and correlations are provided in Table 2.

**Table 2:** Structure-activity relationships of newly synthesized tri-substituted-condensed-imidazopyridines.

Compound	Weight(g/mol)	Hydrogen Bond Acceptors	Net Charge	Hydrogen Bond Donors	SlogP	Surface Area (Å²)	Lipinski Violations
3a	352.7	3	0	0	5.11	323.6	1
3b	374.4	4	0	0	5.11	386.5	1
3c	250.3	3	0	0	3.13	280.6	0
3d	251.3	4	0	0	2.52	273.4	0
3e	280.3	4	0	0	3.14	306.0	0
3f	300.4	3	0	0	4.28	319.7	0
3g	284.7	3	0	0	3.78	294.7	0
3h	355.5	5	0	1	4.13	415.3	0
3i	298.3	4	0	0	3.28	310.4	0
3j	264.3	3	0	0	3.44	293.1	0
3k	318.3	3	0	0	4.46	309.7	0
3l	278.4	3	0	0	3.69	317.1	0
r_spearman_ (IC50)	0.31	-0.19		-0.18	0.43	0.24	

NS: Not Significant

### Statistical analysis

Results were expressed as mean ± SEM of three independent experiments.IC_50_values of individual imidazopyridine derivatives on VRV-PLA_2_-VIII and indirect haemolytic activity were obtained from dose response curve for each derivative.

## Results

### One pot synthesis and characterization of imidazopyridine derivatives

**Fig 2:**
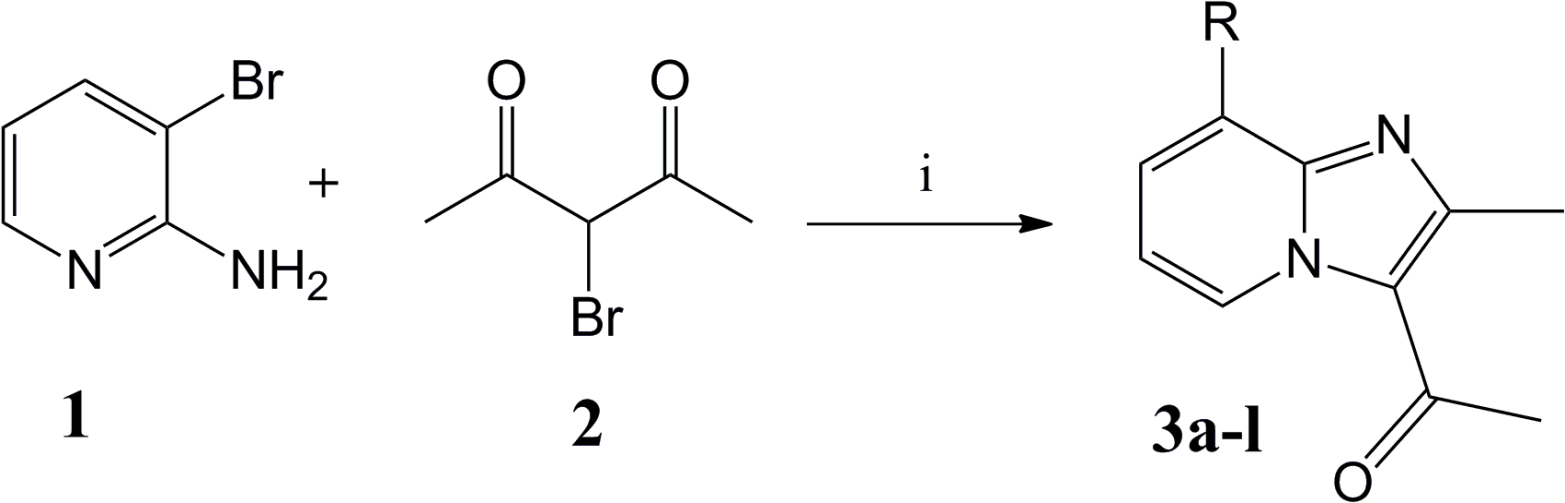
Synthesis of tri-substituted-condensed-imidazopyridines.

We previously reported the newer routes for the synthesis of biologically active heterocycles [24–31] and in continuation we herein report the one pot synthesis of imidazopyridine derivatives. Initially, 3-bromopentane-2,4-dione required for the first step was prepared by treating acetyl acetone with N-Bromosuccinimide (NBS) in chloroform, instead of hazardous molecular bromine (Fig 2). The filtrate of the brominated compound was used directly in the first step without purification, which renders it practically close to the one-step synthesis of the title compounds. The ^1^H NMR spectrum of bromo-derivative of imidazopyridines showed a CH proton at 10 ppm confirming the formation of imidazopyridine ring. In conclusion, we have developed a successful one pot two-step synthesis of Suzuki coupled imidazopyridine derivatives in THF solvent.

### Cheminformatics based rationalization of putative human targets for imidazopyridines

The newly synthesized imidazopyridines were subjected to *in silico* target prediction protocols, which are able to anticipate the most likely protein targets of a small molecule, based on molecular structure. Using the well-established Laplacian-modified Naïve Bayes classifier, *in silico* target prediction of bioactive molecules was carried out based on 155,000 ligand-protein pairs covering 894 human protein targets from ChEMBL.

Among the predicted human targets, B2 bradykinin receptor, cGMP-dependent 3',5'-cyclic phosphodiesterase, Type-2 angiotensin II receptor, Phospholipase A_2_ and TGF-beta receptor type-1 were found to have likelihood scores of 11.54, 9.32, 8.8, 8.14 and 8.05 respectively, which were all empirically classified as being significant. Evidently, imidazopyridines were reported as potent inhibitors for leukotriene A_4_ hydrolase, a pro-inflammatory mediator implicated in the pathogenesis of a number of diseases including inflammatory bowel disease and arthritis [32].Therefore, we considered the Phospholipase A_2_ as a predictive target for the novel imidazopyridines.

### Effect of imidazopyridines towards the inhibitory activity of PLA_2_

**Fig 3:**
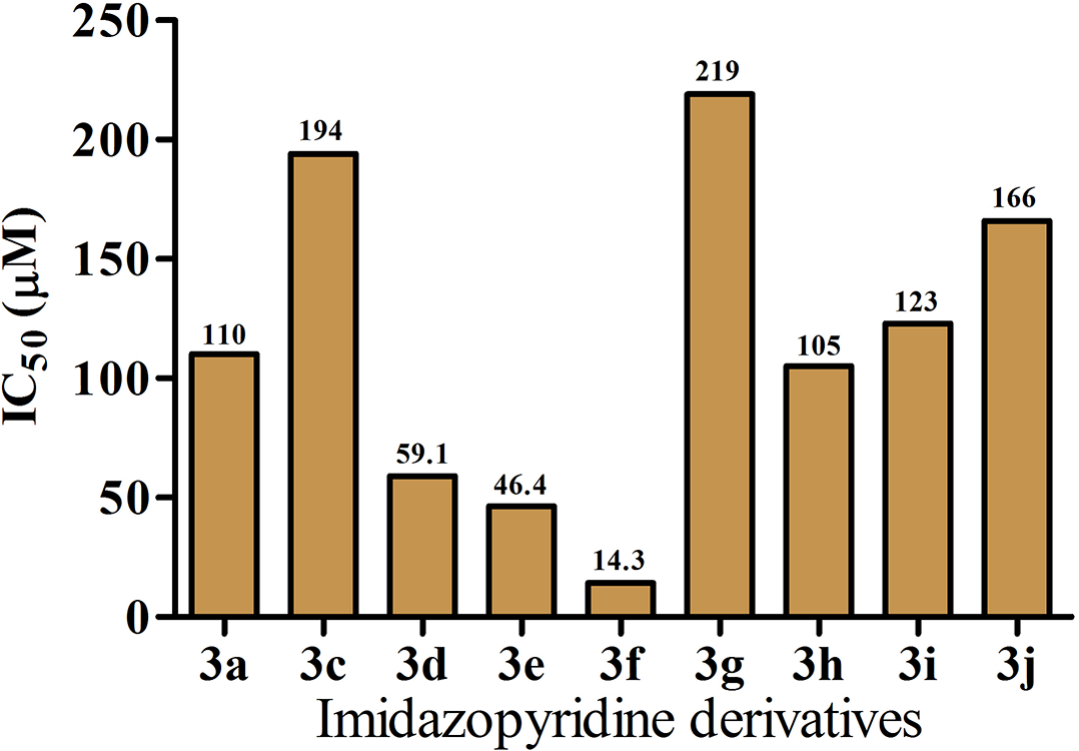
IC_50_ values of imidazopyridine derivatives on *Vipera russelli* (RV) venom induced indirect haemolytic activity. RV venom (1 μg) was pre-incubated with different concentrations of imidazopyridine derivatives for 10 min at 37°C. Assay was performed as described in methods section and IC_**50**_ values for individual imidazopyridine derivatives obtained from dose response curve is presented.

The *in silico* analysis predicted PLA_2_ as a target for the newly synthesized imidazopyridines and we hence tested the imidazopyridines against RV venom PLA_2_ as the target enzyme[20, 33].The series of tri-substituted-condensed-imidazopyridines **3a-l** were assessed for PLA_2_ inhibition by indirect haemolytic activity and the results are tabulated in Table 1. All the tested compounds from tri-substituted-condensed-imidazopyridine series displayed venom-PLA_2_ inhibition in the dose dependent manner. Among the tested compounds, **3f** showed maximum inhibitory efficacy against PLA_2_ with an IC_50_ value of 14.3 μM (Fig 3). None of the compounds induce haemolysis up to the tested concentrations which served as negative control. Additionally, we have tested the effect of imidazopyridines against purified VRV-PLA_2_-VIII, using 1,2-bis(heptanoylthio)glycerophosphocholine as the substrate. The results are summarized in Table 1. The analysis of the results indicated that the imidazopyridines inhibited the catalytic activity of VRV-PLA_2_-VIII, whose IC_50_ values are comparable to the results of indirect haemolytic assay. These results further confirms that imidazopyridines catalytically inhibits VRV-PLA_2_-VIII effectively.

### Structure-based *in silico* docking analysis of imidazopyridine small molecule that targets PLA_2_

To structurally understand the molecular mechanism of inhibition by imidazopyridines, docking studies of imidazopyridines and PLA_2_ were performed. We chose an X-ray structure of Russell's viper PLA_2_ in complex with nimesulide (PDB: 1ZWP) as basis for our docking studies [20]. We docked all 12imidazopyridines (**3a-l**) using MOE to the active site of PLA_2_, thereby replacing the co-crystallized ligand nimesulide.

**Fig 4:**
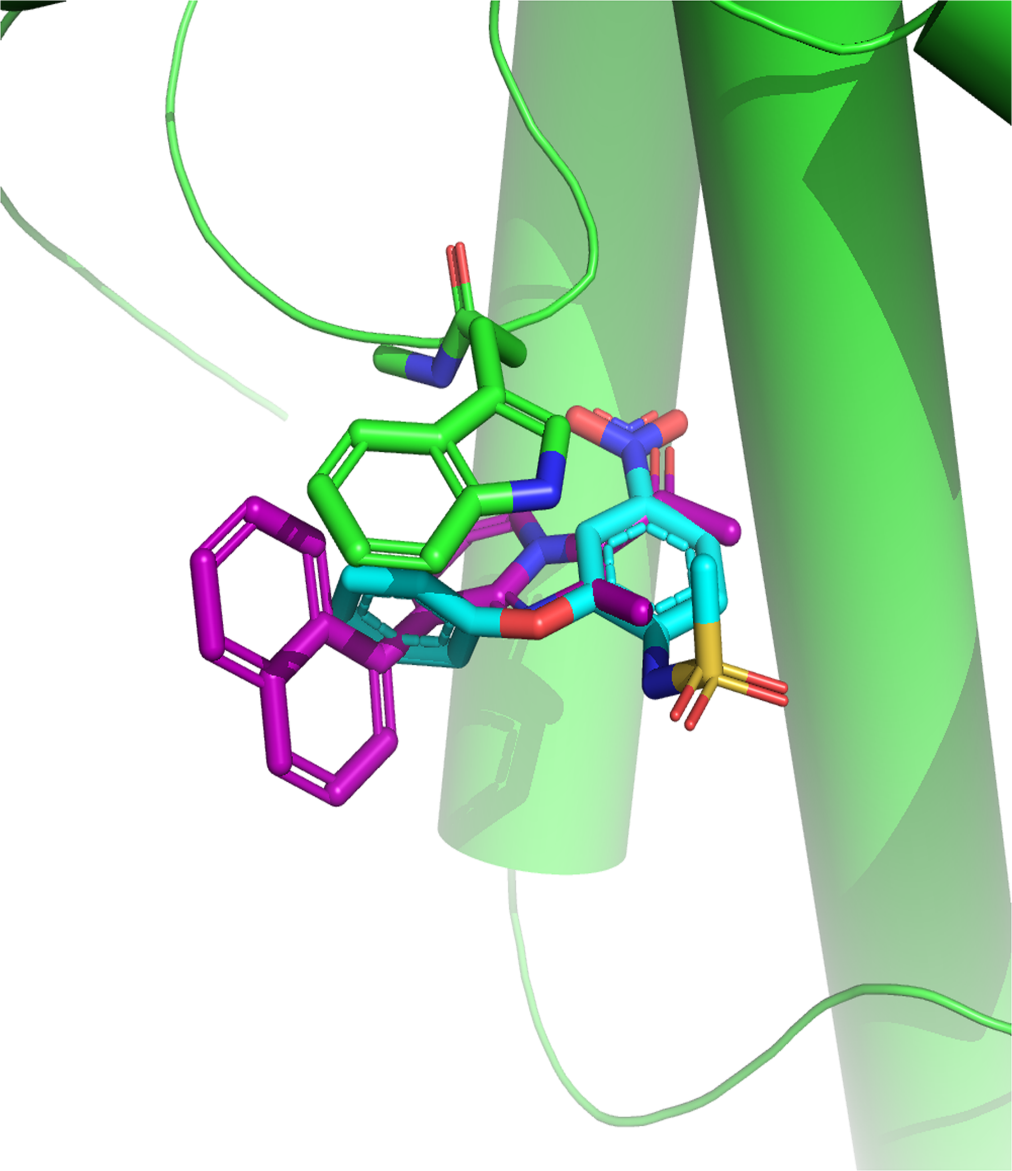
Predicted interactions of imidazopyridine 3f with PLA_2_: PLA_2_ is shown as green cartoon with highlighted Trp-31 and peptide bond to Gly-32. Co-crystallized ligand nimesulide is shown in stick representation with cyan carbon atoms. The highest scoring docking pose of compound **3f** is shown in purple sticks. *In silico* docking predicts π-π stacking interactions with Trp-31 and additional amide-π interactions with the backbone of Gly-32.

All the docked compounds occupy a similar region in the PLA_2_ binding site, thereby replacing the nitro group of nimesulide with either a ketone or an ether functionality. The position of the phenyl ring of nimesulide is occupied by the imidazopyridine for most predicted poses; thereby showing pronounced π-π stacking interactions with Trp-31 (Fig 4). The most active compound, imidazopyridine **3f**, ranks second amongst the twelve docked compounds. The naphthyl system of **3f** forms additional stacking interactions with Trp-31 and extends towards Gly-32 potentially adding further amide-pi stacking contributions. Therefore, molecular docking studies were found helpful in rationalization of PLA_2_ *in vitro* binding.

### Structure-activity relationships

We found only modest correlations between physico-chemical descriptors and experimental bioactivity. Interestingly, we observe weak correlations in our compound set indicating that high molecular weight and high lipophilicity reduce PLA_2_ binding. Only two of twelve compounds (**3a, 3b**) show a single violation of Lipinski's Rule of Five due to a calculated logP of 5.11. Therefore, the compounds are predicted to be orally bioavailable.

## Discussion

The imidazopyridine scaffold has been extensively incorporated in many drugs because of its medicinal properties over the other heterocyclic cores. In the present work, we developed a new method to prepare imidazopyridine-based compounds without the use of catalyst for cyclization. Additionally, replacement of halogen atom with desired moiety in the title compounds provides a platform for the derivatization and diversification of imidazopyridines. On the other hand, secretory PLA_2s_ are ubiquitous in mammalian tissues as well as animal venom. PLA_2s_ are the lipolytic enzymes with the ability to catalyze the hydrolysis of sn-2 ester bonds in a variety of glycerophospholipid molecules releasing fatty acids and lysophospholipids [34, 35]. The catabolic products of glycerophospholipids are known to be the mediators of various inflammatory diseases [36]. In this study, snake venom PLA_2_ was used as a model enzyme to study the inhibitory efficacy of newly synthesized tri-substituted-condensed-imidazopyridines.

## Conclusion

In conclusion, we herein report a simple, efficient, catalyst free and one pot synthetic route to prepare tri-substituted-condensed-imidazopyridines and our *in silico* target prediction presented PLA_2_ as a likely target for the newly synthesized compounds. The prediction was experimentally validated using VRV-PLA_2_-VIII and indirect haemolytic assay. Of the new compounds synthesized, 1-(2-Methyl-8-naphthalen-1-yl-imidazo [1,2-α]pyridine-3-yl)-ethanone was identified as the lead compound with an IC_50_ value of 14.3 μM. Molecular docking analysis displayed that the imidazopyridine compounds could make a favourable π-π stacking interactions with Trp-31. Exploration of PLA_2_ inhibitory activity of imidazopyridine derivatives contributes to the development of the title compounds as therapeutic agents to block the PLA_2_ associated inflammatory diseases. Thus, synthesis of more imidazopyridine derivatives and optimization of their biological activity according to the identified structure-activity relationship is envisaged.

## Supporting Information

S1 Data Scanned spectral images of novel imidazopyridine derivatives. (DOCX)

S2 Data Structural analysis of novel imidazopyridine derivative. (DOCX)

## References

[1] El Kazzouli S, Griffon du Bellay A, Berteina-Raboin S, Delagrange P, Caignard DH, Guillaumet G. Design and synthesis of 2-phenylimidazo[1,2-a]pyridines as a novel class of melatonin receptor ligands. European journal of medicinal chemistry. 2011;46(9):4252–7. Epub 2011/07/19. 10.1016/j.ejmech.2011.06.030 . 21764185

[2] Martinez-Urbina MA, Zentella A, Vilchis-Reyes MA, Guzman A, Vargas O, Ramirez Apan MT, et al 6-Substituted 2-(N-trifluoroacetylamino)imidazopyridines induce cell cycle arrest and apoptosis in SK-LU-1 human cancer cell line. European journal of medicinal chemistry. 2010;45(3):1211–9. Epub 2010/01/05. 10.1016/j.ejmech.2009.11.049 . 20045224

[3] Al-Tel TH, Al-Qawasmeh RA, Zaarour R. Design, synthesis and in vitro antimicrobial evaluation of novel Imidazo[1,2-a]pyridine and imidazo[2,1-b][1,3]benzothiazole motifs. European journal of medicinal chemistry. 2011;46(5):1874–81. Epub 2011/03/19. 10.1016/j.ejmech.2011.02.051 . 21414694

[4] Kamal A, Reddy JS, Ramaiah MJ, Dastagiri D, Bharathi EV, Sagar MVP, et al Design, synthesis and biological evaluation of imidazopyridine/pyrimidine-chalcone derivatives as potential anticancer agents. MedChemComm. 2010;1(5):355–60.

[5] Reddy BS, Reddy PS, Reddy YJ, Yadav J. InBr 3-catalyzed three-component, one-pot synthesis of imidazo [1, 2-a] pyridines. Tetrahedron Letters. 2011;52(44):5789–93.

[6] Kamal A, Ramakrishna G, Raju P, Rao AV, Viswanath A, Nayak VL, et al Synthesis and anticancer activity of oxindole derived imidazo[1,5-a]pyrazines. European journal of medicinal chemistry. 2011;46(6):2427–35. Epub 2011/04/13. 10.1016/j.ejmech.2011.03.027 . 21481986

[7] Chernyak N, Gevorgyan V. General and Efficient Copper‐Catalyzed Three‐Component Coupling Reaction towards Imidazoheterocycles: One‐Pot Synthesis of Alpidem and Zolpidem. Angewandte Chemie International Edition. 2010;49(15):2743–6. 20213787 10.1002/anie.200907291 PMC3516864

[8] Palani T, Park K, Kumar MR, Jung HM, Lee S. Copper‐Catalyzed Decarboxylative Three‐Component Reactions for the Synthesis of Imidazo [1, 2‐a] pyridines. European Journal of Organic Chemistry. 2012;2012(26):5038–47.

[9] Chioua M, Soriano E, Infantes L, Jimeno ML, Marco-Contelles J, Samadi A. Silver-Catalyzed Cyclization of N-(Prop-2-yn-1-yl)pyridin-2-amines. European Journal of Organic Chemistry. 2013;2013(1):35–9. 10.1002/ejoc.201201258

[10] Bakherad M, Nasr-Isfahani H, Keivanloo A, Doostmohammadi N. Pd–Cu catalyzed heterocyclization during Sonogashira coupling: synthesis of 2-benzylimidazo [1, 2-a] pyridine. Tetrahedron Letters. 2008;49(23):3819–22.

[11] DiMauro EF, Vitullo JR. Microwave-Assisted Preparation of Fused Bicyclic Heteroaryl Boronates: Application in One-Pot Suzuki Couplings. The Journal of Organic Chemistry. 2006;71(10):3959–62. 10.1021/jo060218p 16674073

[12] Lamblin M, Nassar‐Hardy L, Hierso JC, Fouquet E, Felpin FX. Recyclable heterogeneous palladium catalysts in pure water: Sustainable developments in Suzuki, Heck, Sonogashira and Tsuji–Trost reactions. Advanced Synthesis & Catalysis. 2010;352(1):33–79.

[13] Jayanthi GP, Kasturi S, Gowda TV. Dissociation of catalytic activity and neurotoxicity of a basic phospholipase A2 from Russell's viper (Vipera russelli) venom. Toxicon: official journal of the International Society on Toxinology. 1989;27(8):875–85. Epub 1989/01/01. . 2781586 10.1016/0041-0101(89)90099-8

[14] van der Horst E, Peironcely JE, van Westen GJ, van den Hoven OO, Galloway WR, Spring DR, et al Chemogenomics approaches for receptor deorphanization and extensions of the chemogenomics concept to phenotypic space. Current topics in medicinal chemistry. 2011;11(15):1964–77. Epub 2011/04/08. . 21470175 10.2174/156802611796391230

[15] Koutsoukas A, Lowe R, Kalantarmotamedi Y, Mussa HY, Klaffke W, Mitchell JB, et al In silico target predictions: defining a benchmarking data set and comparison of performance of the multiclass Naive Bayes and Parzen-Rosenblatt window. Journal of chemical information and modeling. 2013;53(8):1957–66. Epub 2013/07/09. 10.1021/ci300435j . 23829430

[16] Koutsoukas A, Simms B, Kirchmair J, Bond PJ, Whitmore AV, Zimmer S, et al From in silico target prediction to multi-target drug design: current databases, methods and applications. Journal of proteomics. 2011;74(12):2554–74. Epub 2011/05/31. 10.1016/j.jprot.2011.05.011 . 21621023

[17] Gaulton A, Bellis LJ, Bento AP, Chambers J, Davies M, Hersey A, et al ChEMBL: a large-scale bioactivity database for drug discovery. Nucleic acids research. 2012;40(Database issue):D1100–7. Epub 2011/09/29. 10.1093/nar/gkr777 ; PubMed Central PMCID: PMCPmc3245175. 21948594 PMC3245175

[18] Boman HG, Kaletta U. Chromatography of rattlesnake venom; a separation of three phosphodiesterases. Biochimica et biophysica acta. 1957;24(3):619–31. Epub 1957/06/01. . 13436488 10.1016/0006-3002(57)90256-1

[19] Petrovic N, Grove C, Langton PE, Misso NL, Thompson PJ. A simple assay for a human serum phospholipase A2 that is associated with high-density lipoproteins. Journal of lipid research. 2001;42(10):1706–13. Epub 2001/10/09. . 11590228

[20] Singh N, Kumar RP, Kumar S, Sharma S, Mir R, Kaur P, et al Simultaneous inhibition of anti-coagulation and inflammation: crystal structure of phospholipase A2 complexed with indomethacin at 1.4 A resolution reveals the presence of the new common ligand-binding site. Journal of molecular recognition: JMR. 2009;22(6):437–45. Epub 2009/05/23. 10.1002/jmr.960 . 19462410

[21] Molecular Operating Environment (MOE) CCGI, Montreal Q, Canada, 2013.

[22] Naim M, Bhat S, Rankin KN, Dennis S, Chowdhury SF, Siddiqi I, et al Solvated interaction energy (SIE) for scoring protein-ligand binding affinities. 1. Exploring the parameter space. Journal of chemical information and modeling. 2007;47(1):122–33. Epub 2007/01/24. 10.1021/ci600406v . 17238257

[23] De Lano W: The Pymol Molecular Graphics System v, De Lano Scientific, San Carlos, CA.

[24] Sugahara K, Thimmaiah KN, Bid HK, Houghton PJ, Rangappa KS. Anti-tumor activity of a novel HS-mimetic-vascular endothelial growth factor binding small molecule. PloS one. 2012;7(8):e39444 Epub 2012/08/24. 10.1371/journal.pone.0039444 ; PubMed Central PMCID: PMCPmc3419744. 22916091 PMC3419744

[25] Kumar CA, Jayarama S, Salimath BP, Rangappa KS. Pro-apoptotic activity of imidazole derivatives mediated by up-regulation of Bax and activation of CAD in Ehrlich Ascites Tumor cells. Investigational new drugs. 2007;25(4):343–50. 17372679 10.1007/s10637-006-9033-4

[26] Keerthy HK, Garg M, Mohan CD, Madan V, Kanojia D, Shobith R, et al Synthesis and characterization of novel 2-amino-chromene-nitriles that target Bcl-2 in acute myeloid leukemia cell lines. PloS one. 2014;9(9):e107118 10.1371/journal.pone.0107118 25268519 PMC4182326

[27] Keerthy HK, Mohan CD, Siveen KS, Fuchs JE, Rangappa S, Sundaram MS, et al Novel synthetic biscoumarins target tumor necrosis factor-α in hepatocellular carcinoma in vitro and in vivo. Journal of Biological Chemistry. 2014;289(46):31879–90. 10.1074/jbc.M114.593855 25231984 PMC4231667

[28] Mohan CD, Bharathkumar H, Bulusu KC, Pandey V, Rangappa S, Fuchs JE, et al Development of a Novel Azaspirane That Targets the Janus Kinase-Signal Transducer and Activator of Transcription (STAT) Pathway in Hepatocellular Carcinoma in Vitro and in Vivo. Journal of Biological Chemistry. 2014;289(49):34296–307. 10.1074/jbc.M114.601104 25320076 PMC4256360

[29] Rakesh KS, Jagadish S, Vinayaka AC, Hemshekhar M, Paul M, Thushara RM, et al A New Ibuprofen Derivative Inhibits Platelet Aggregation and ROS Mediated Platelet Apoptosis. PloS one. 2014;9(9):e107182 10.1371/journal.pone.0107182 25238069 PMC4169656

[30] Fongmoon D, Shetty AK, Basappa, Yamada S, Sugiura M, Kongtawelert P, et al Chondroitinase-mediated degradation of rare 3-O-sulfated glucuronic acid in functional oversulfated chondroitin sulfate K and E. The Journal of biological chemistry. 2007;282(51):36895–904. Epub 2007/10/24. 10.1074/jbc.M707082200 . 17951579

[31] Anusha S, Anandakumar BS, Mohan CD, Nagabhushana GP, Priya BS, Rangappa KS, et al Preparation and use of combustion-derived Bi2O3 for the synthesis of heterocycles with anti-cancer properties by Suzuki-coupling reactions. RSC Advances. 2014;4(94):52181–8. 10.1039/C4RA07839J

[32] Penning TD, Chandrakumar NS, Desai BN, Djuric SW, Gasiecki AF, Malecha JW, et al Synthesis of imidazopyridines and purines as potent inhibitors of leukotriene A4 hydrolase. Bioorganic & medicinal chemistry letters. 2003;13(6):1137–9. Epub 2003/03/20. . 12643929 10.1016/s0960-894x(03)00039-8

[33] Sunitha K, Hemshekhar M, Gaonkar SL, Sebastin Santhosh M, Suresh Kumar M, Basappa, et al Neutralization of haemorrhagic activity of viper venoms by 1-(3-dimethylaminopropyl)-1-(4-fluorophenyl)-3-oxo-1,3-dihydroisobenzofuran-5-car bonitrile. Basic & clinical pharmacology & toxicology. 2011;109(4):292–9. Epub 2011/07/07. 10.1111/j.1742-7843.2011.00725.x . 21729242

[34] Sekar K, Vaijayanthi Mala S, Yogavel M, Velmurugan D, Poi MJ, Vishwanath BS, et al Crystal structures of the free and anisic acid bound triple mutant of phospholipase A2. Journal of molecular biology. 2003;333(2):367–76. Epub 2003/10/08. . 14529623 10.1016/j.jmb.2003.08.032

[35] Marcussi S, Sant'Ana CD, Oliveira CZ, Rueda AQ, Menaldo DL, Beleboni RO, et al Snake venom phospholipase A2 inhibitors: medicinal chemistry and therapeutic potential. Current topics in medicinal chemistry. 2007;7(8):743–56. Epub 2007/04/26. . 17456038 10.2174/156802607780487614

[36] Frisardi V, Panza F, Seripa D, Farooqui T, Farooqui AA. Glycerophospholipids and glycerophospholipid-derived lipid mediators: a complex meshwork in Alzheimer’s disease pathology. Progress in lipid research. 2011;50(4):313–30. 10.1016/j.plipres.2011.06.001 21703303

