# Supplementary figures and images for: A One Pot Synthesis of Novel Bioactive Tri-Substitute-Condensed-Imidazopyridines that Targets Snake Venom Phospholipase A2
Source: PLoS One. 2015 Jul 21;10(7):e0131896. doi: 10.1371/journal.pone.0131896 (PMC4511007; doi:10.1371/journal.pone.0131896)

**Data S1**

1H NMR

**3a
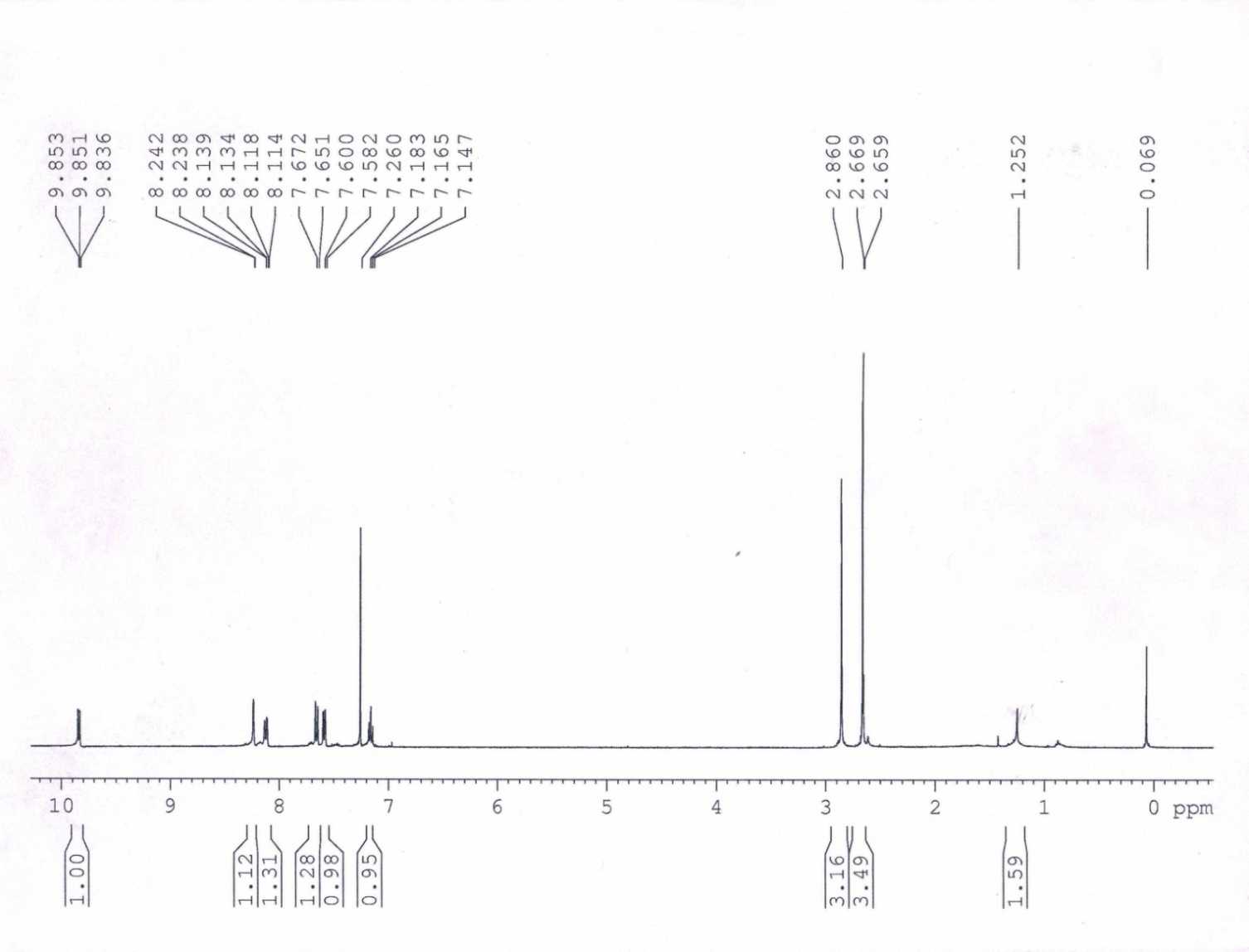
3b** **
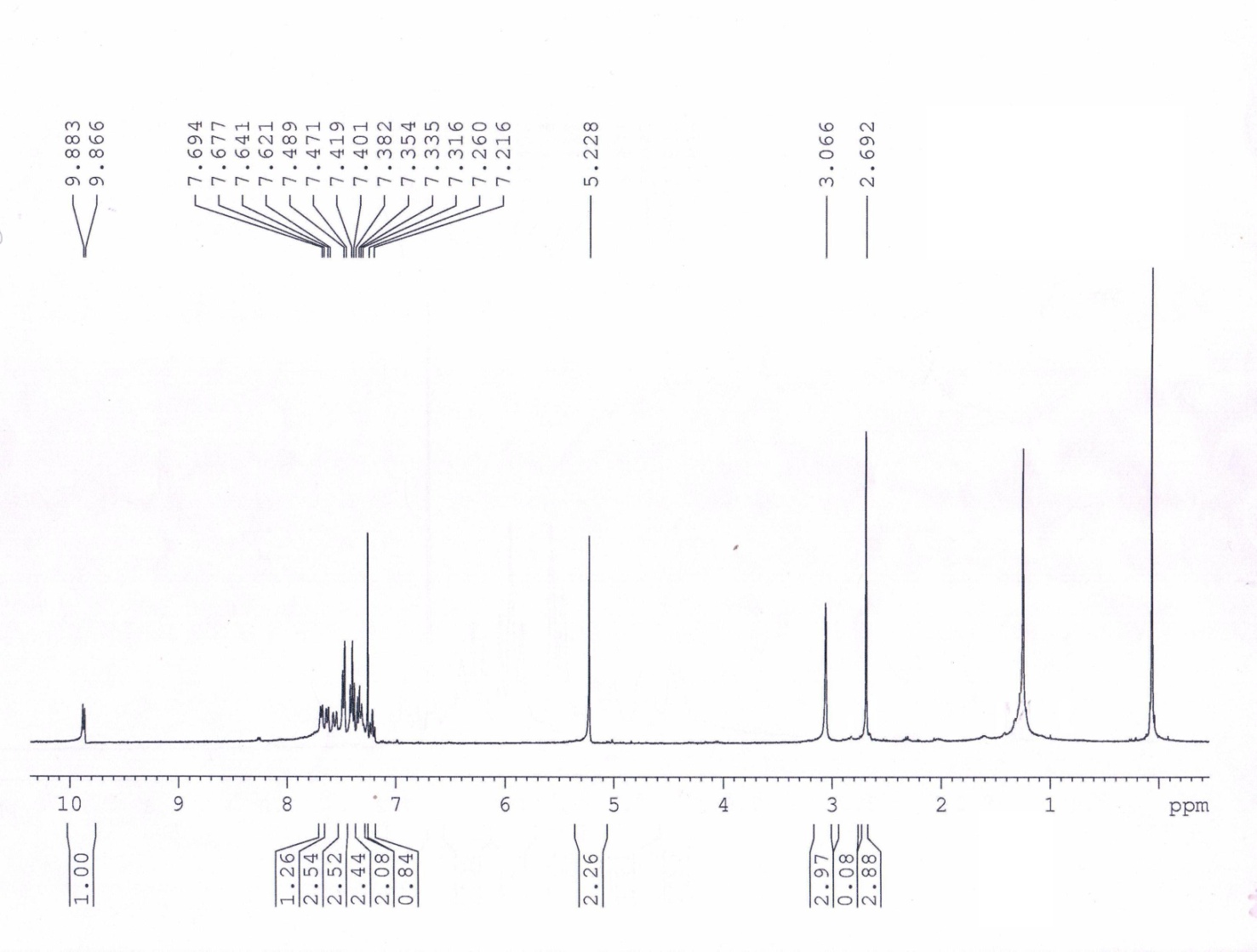
**

**3c**
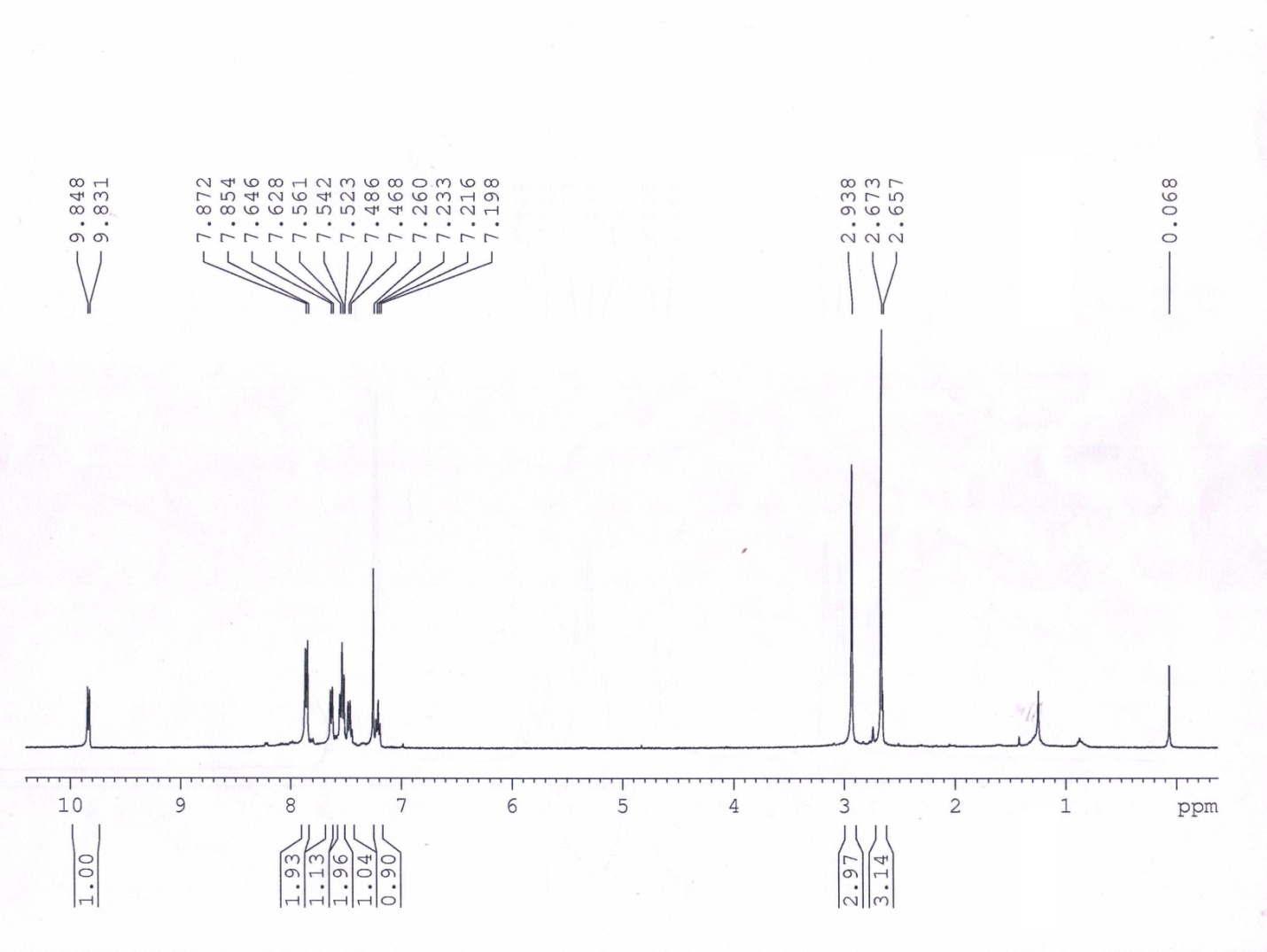


**3d**
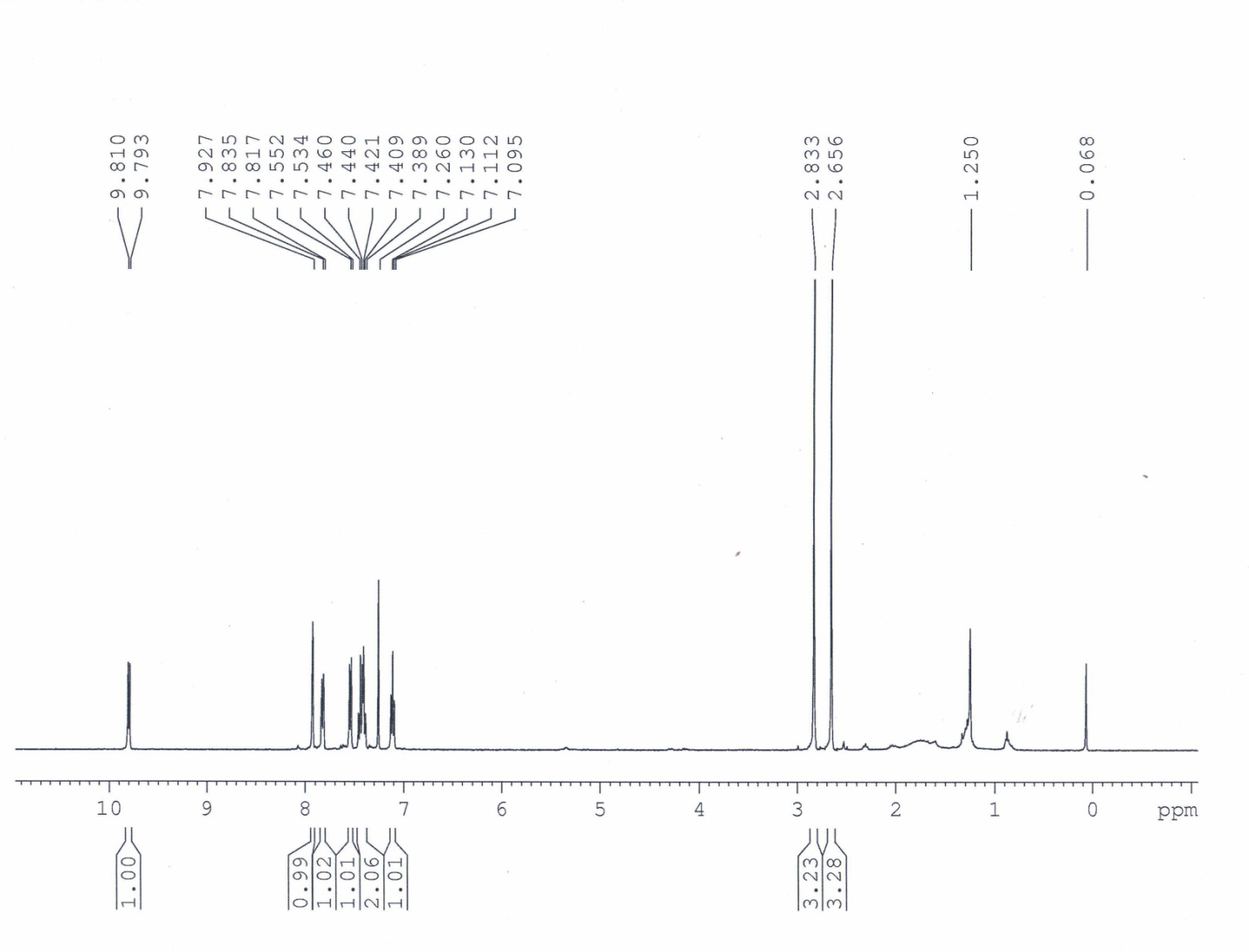


**3e
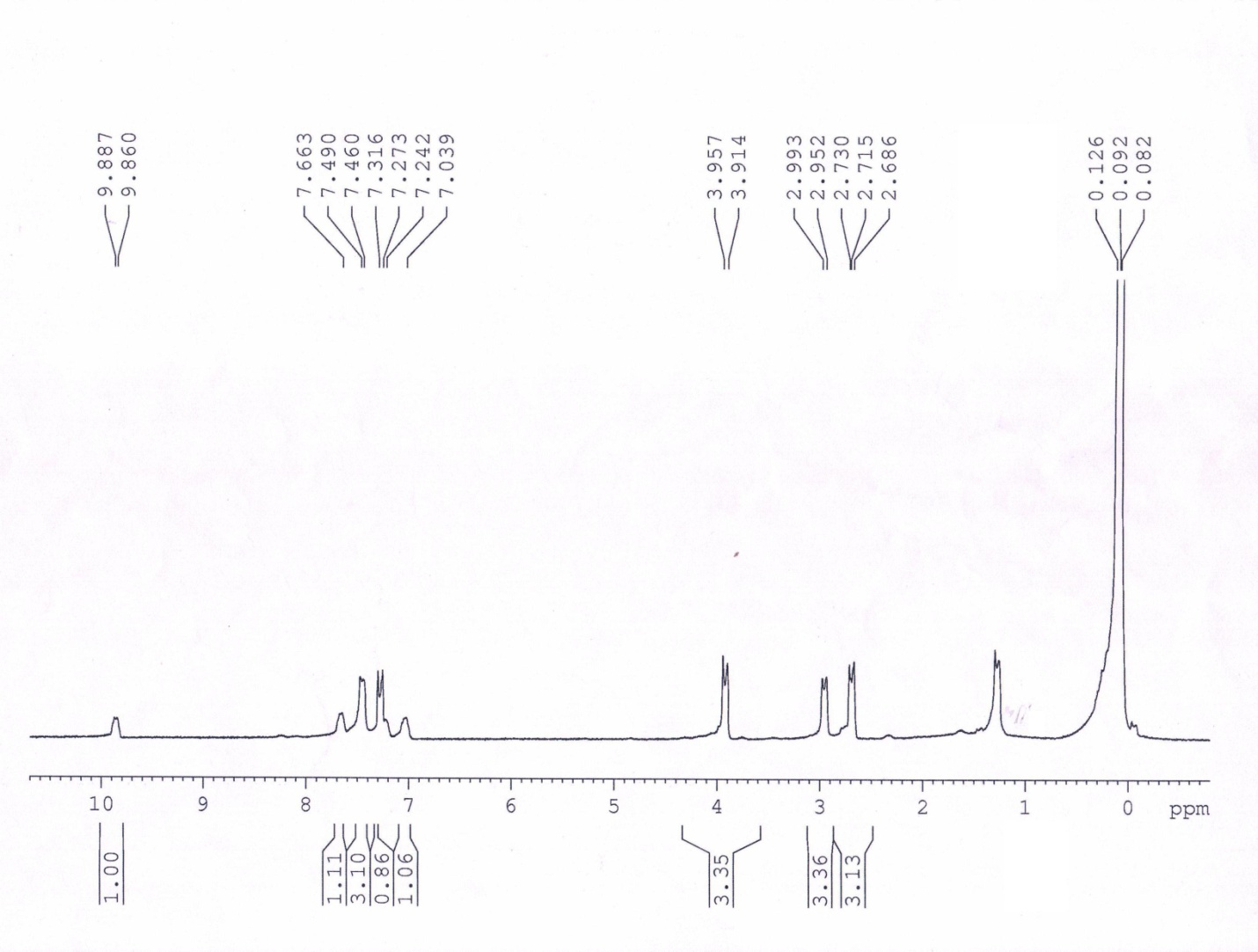
**

**3f**
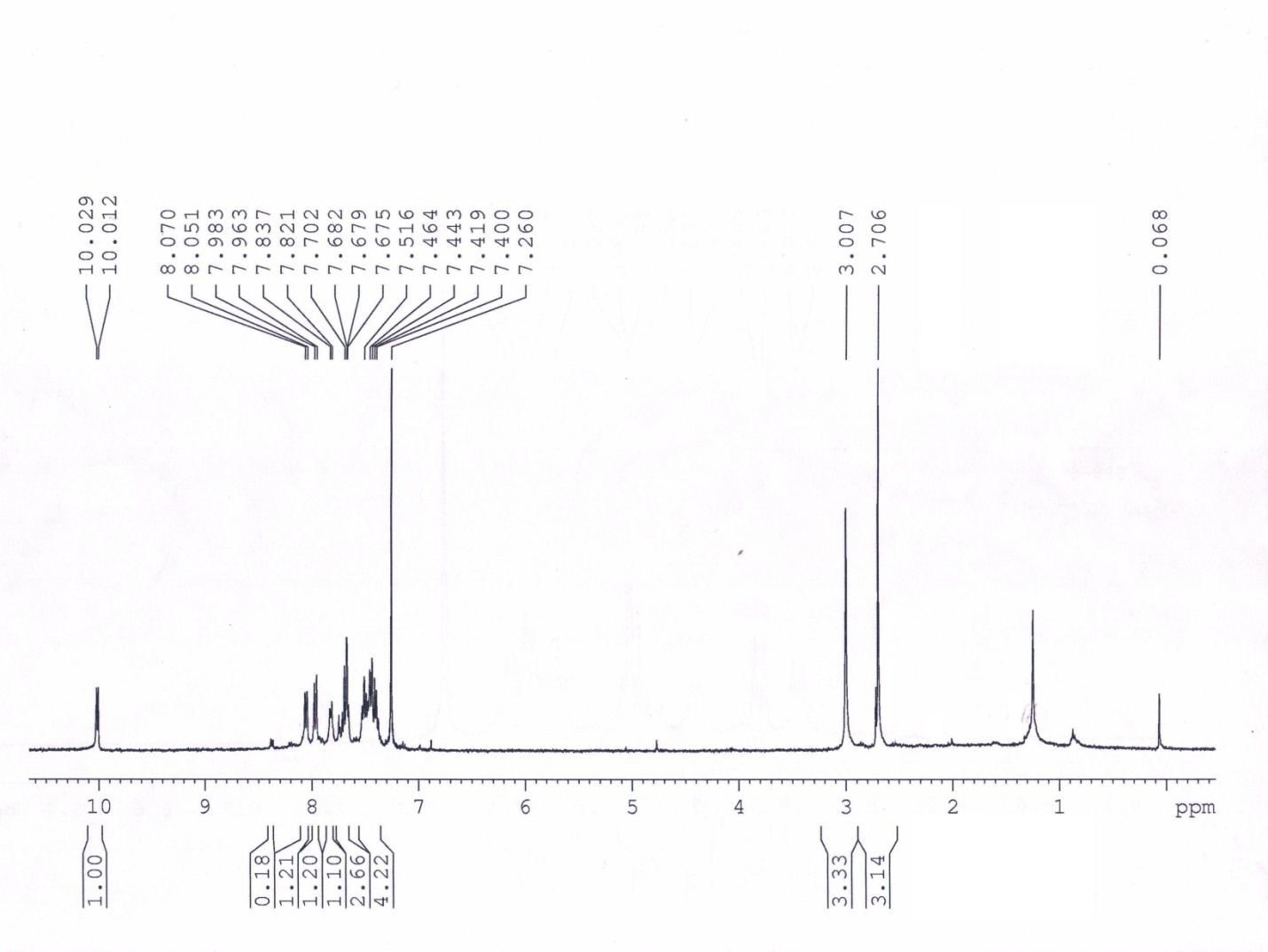


**3g**
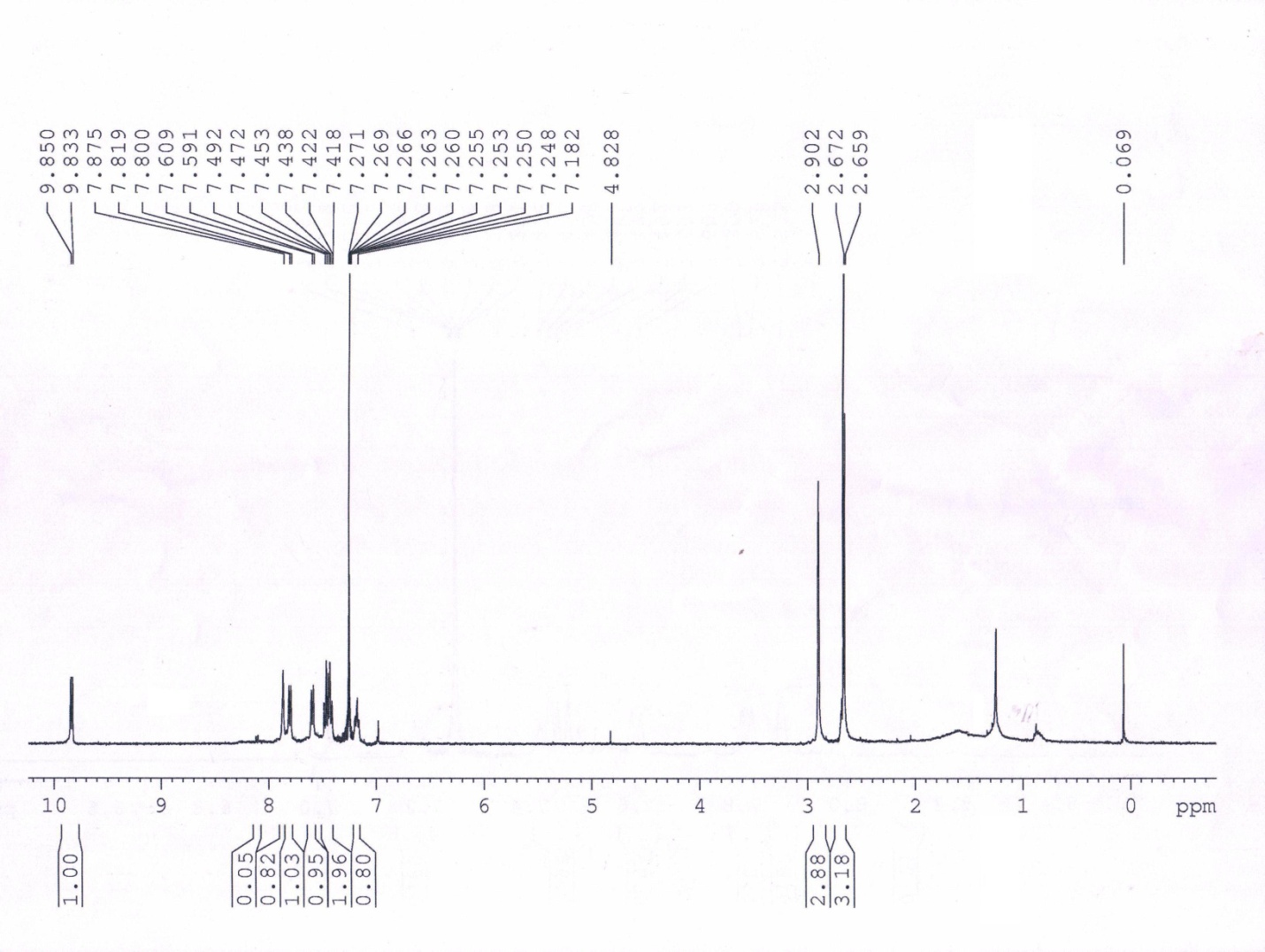


**3h**
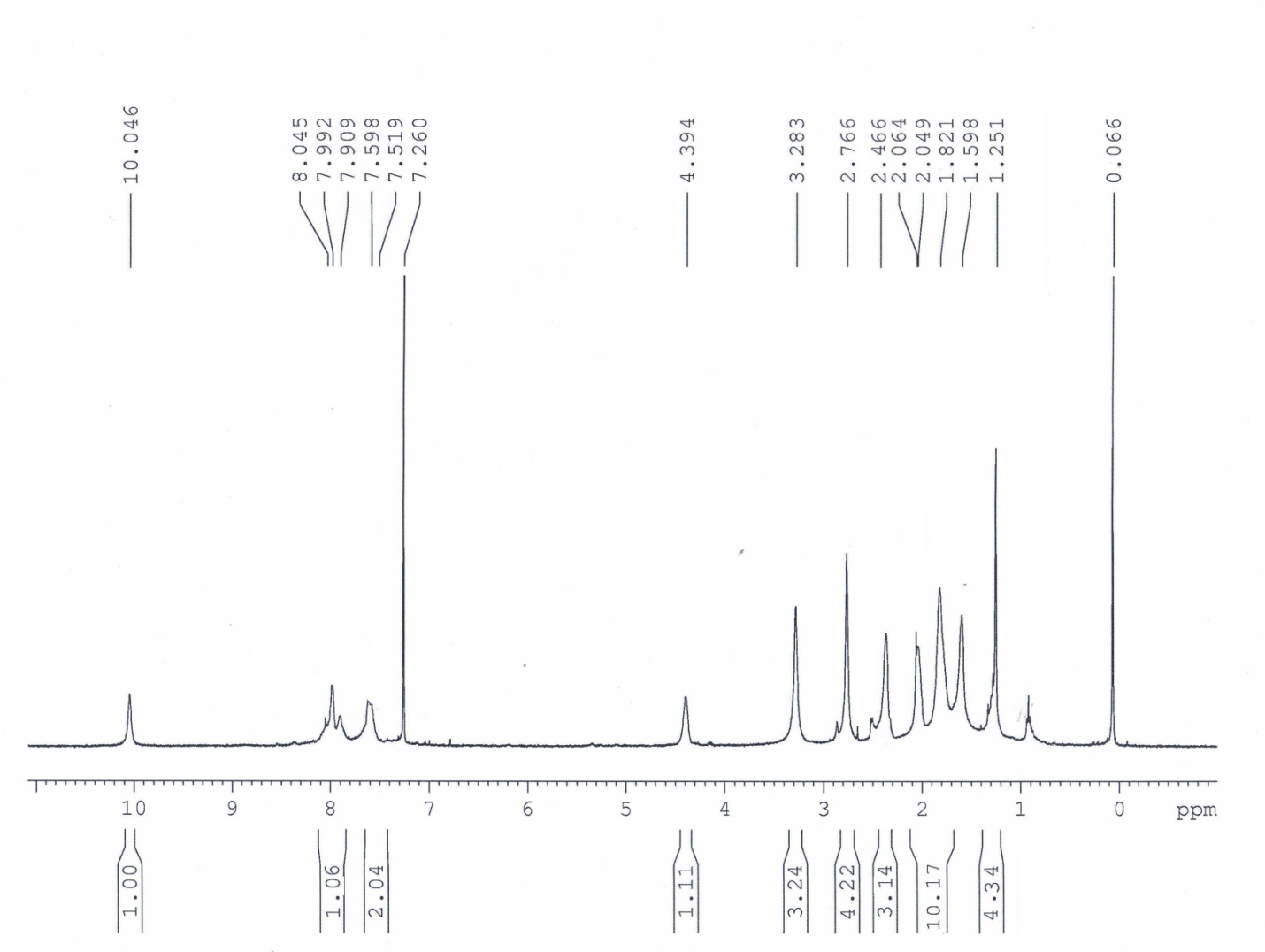
**3i**
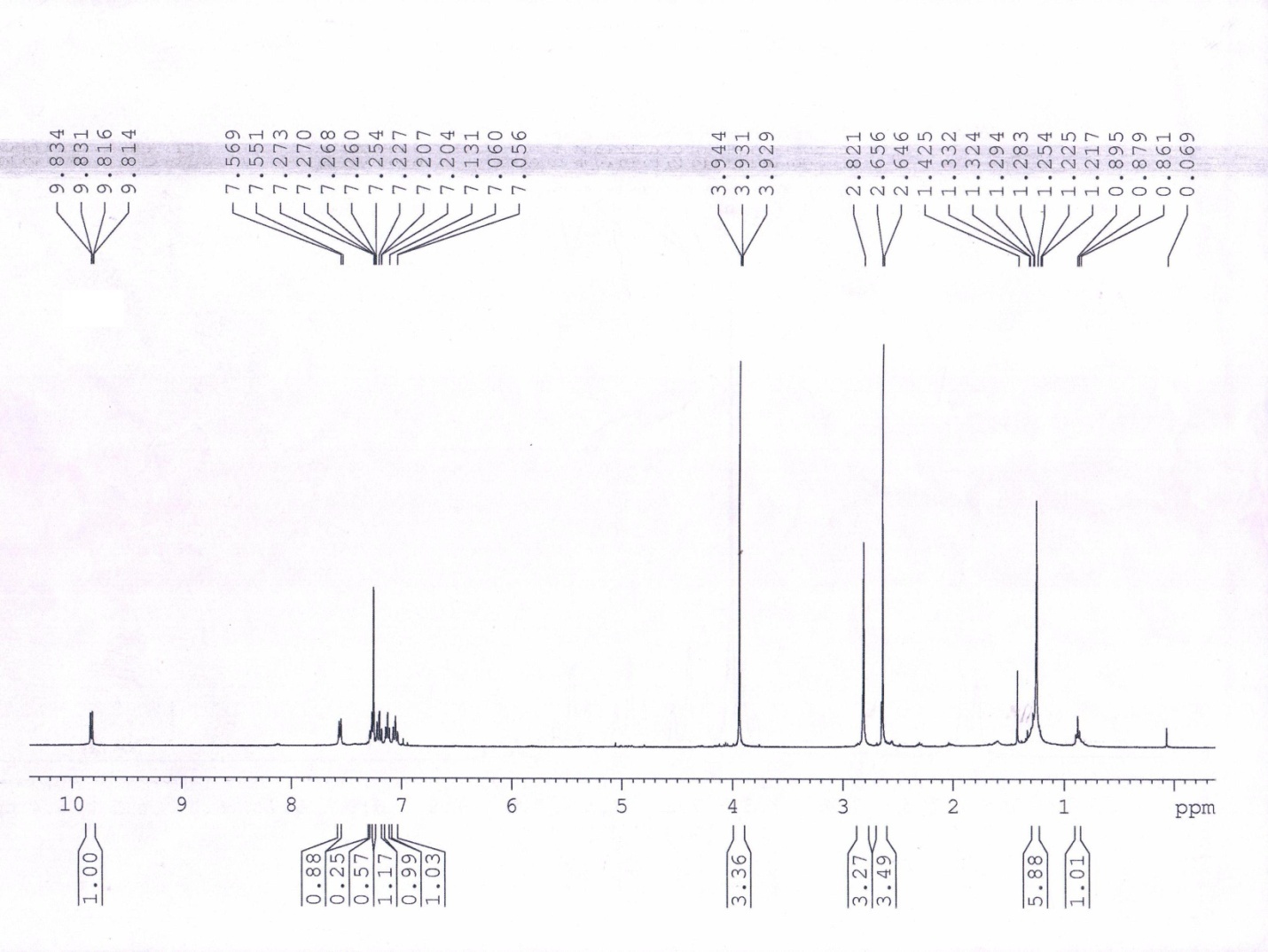


**3j**
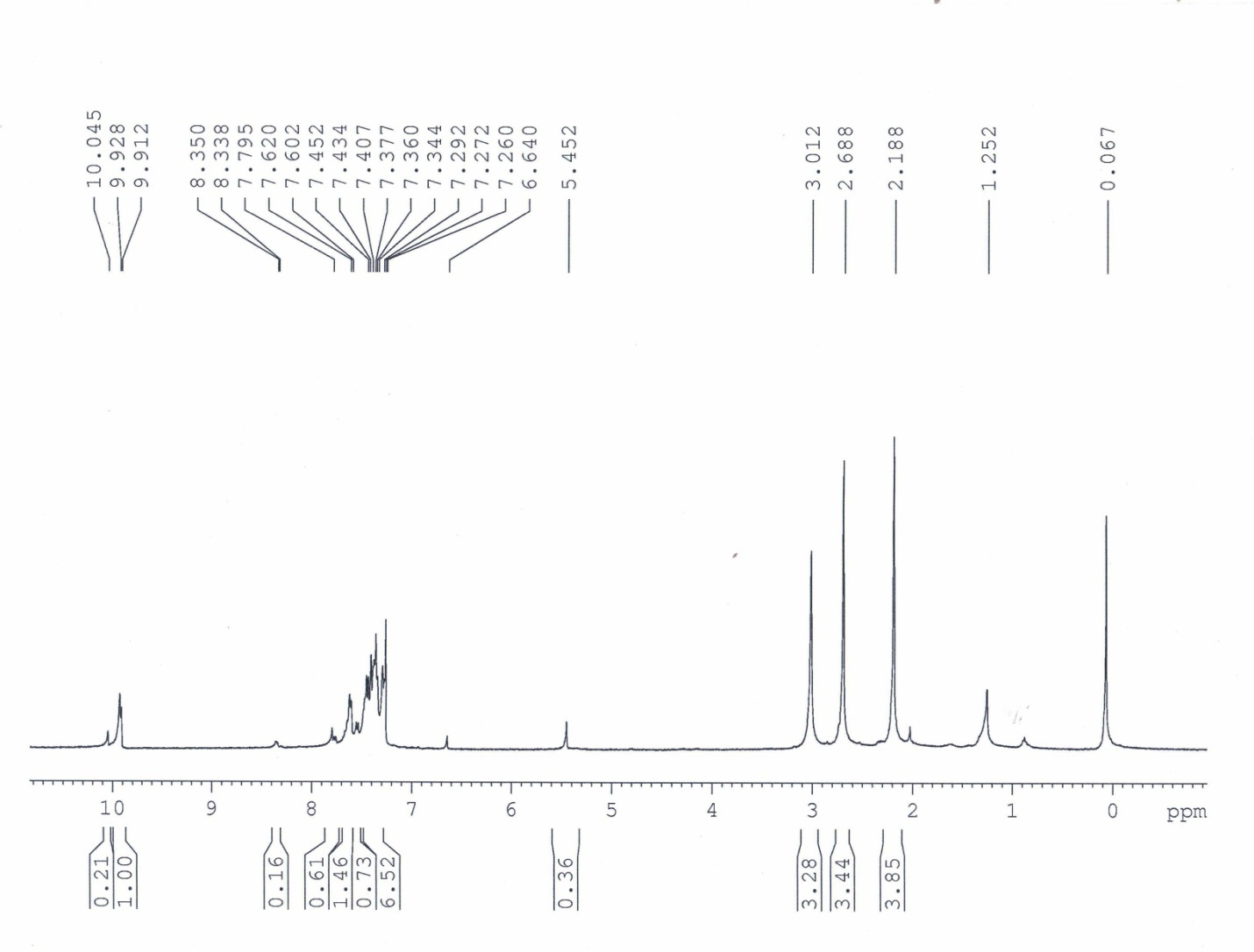


**3k**
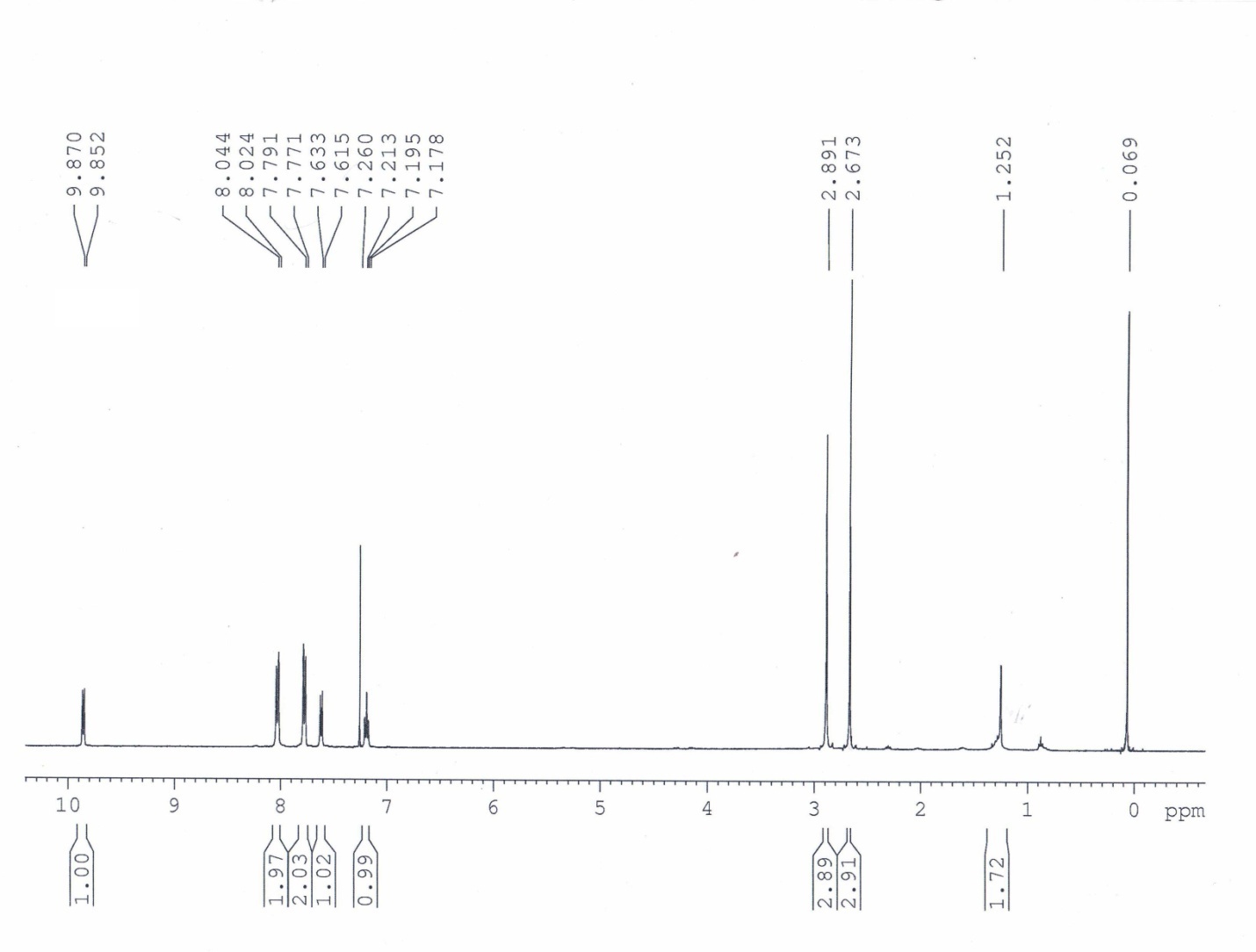


**3l**
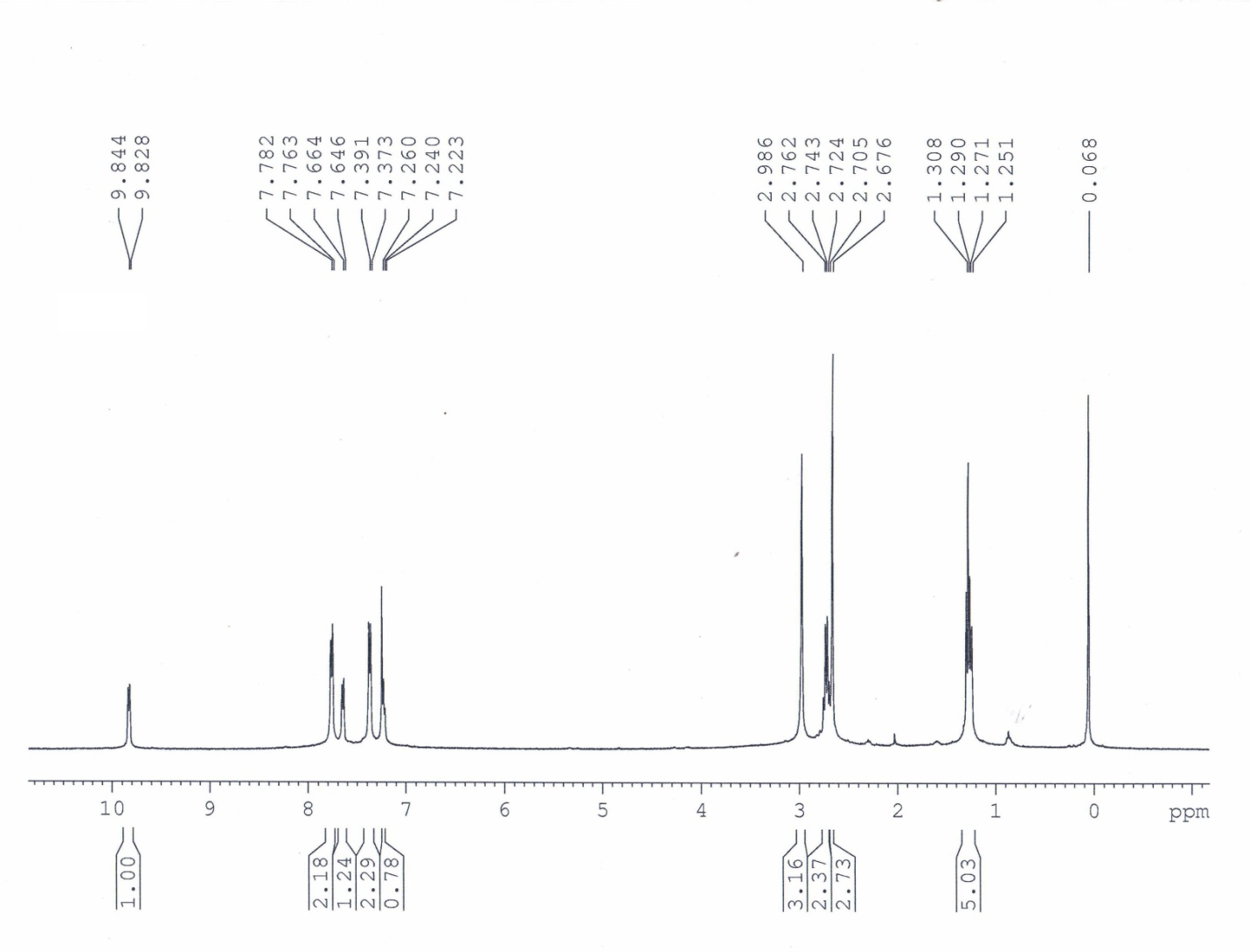


13CNMR

**3a**
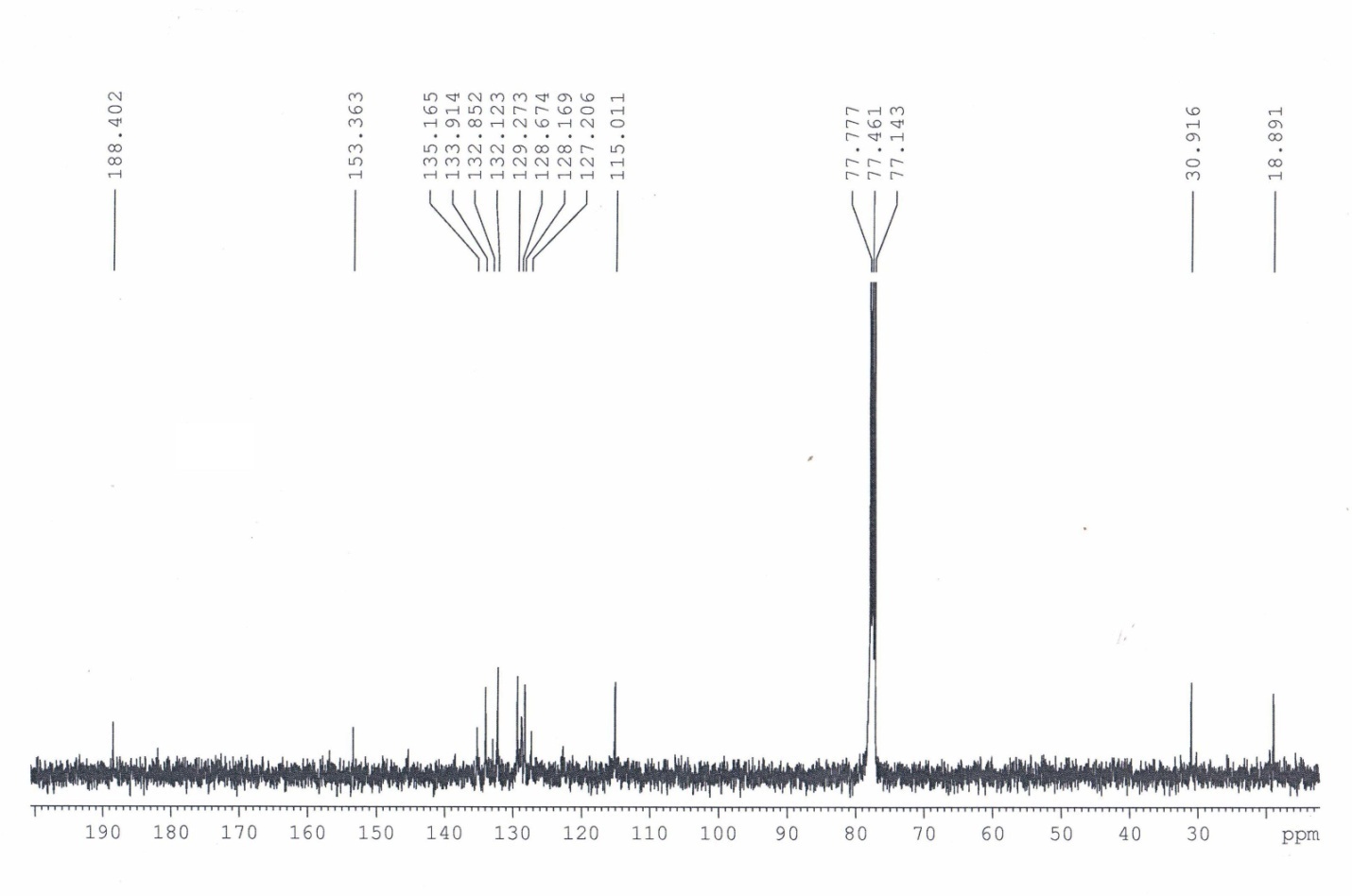


**3b**
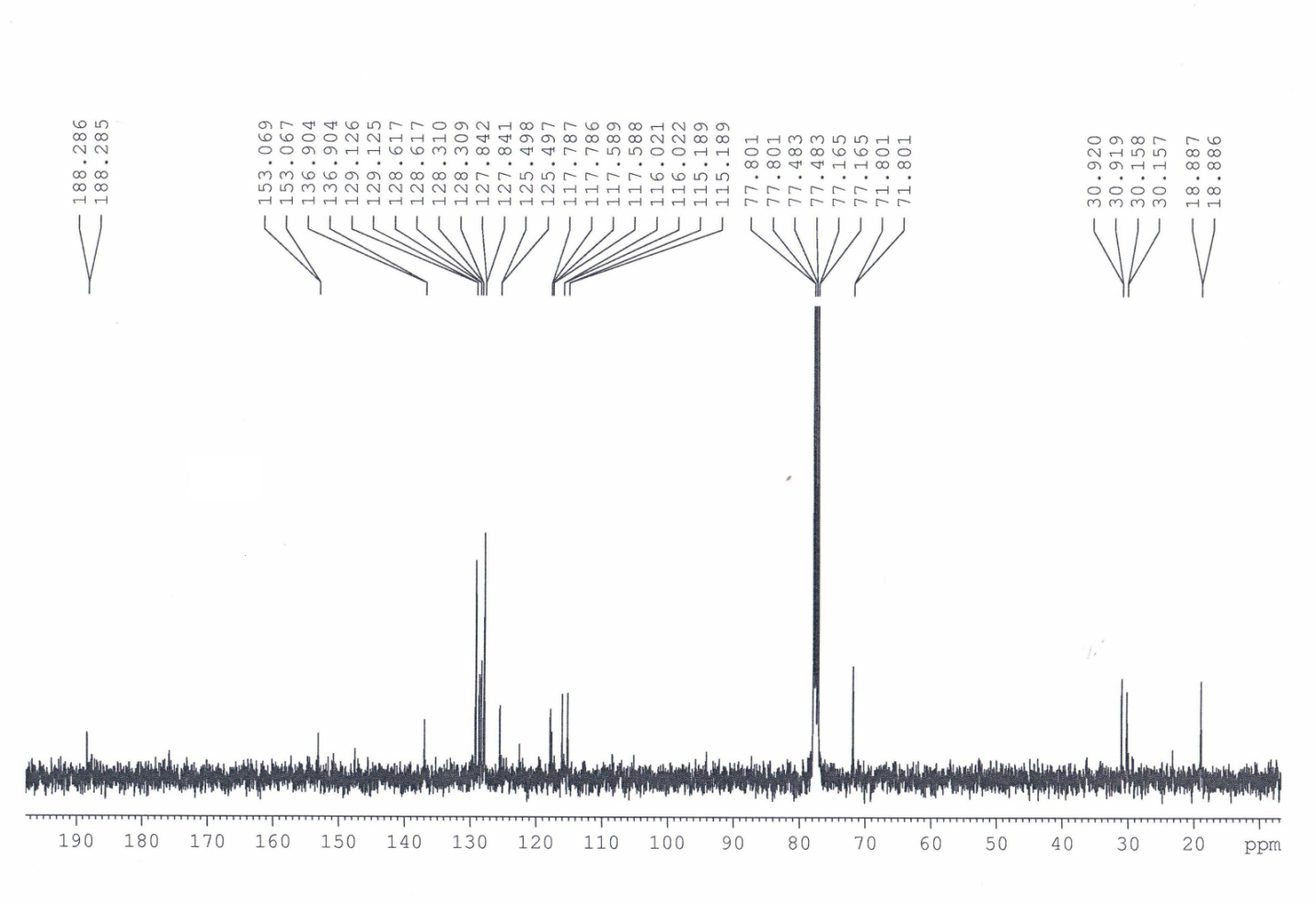


**3e**
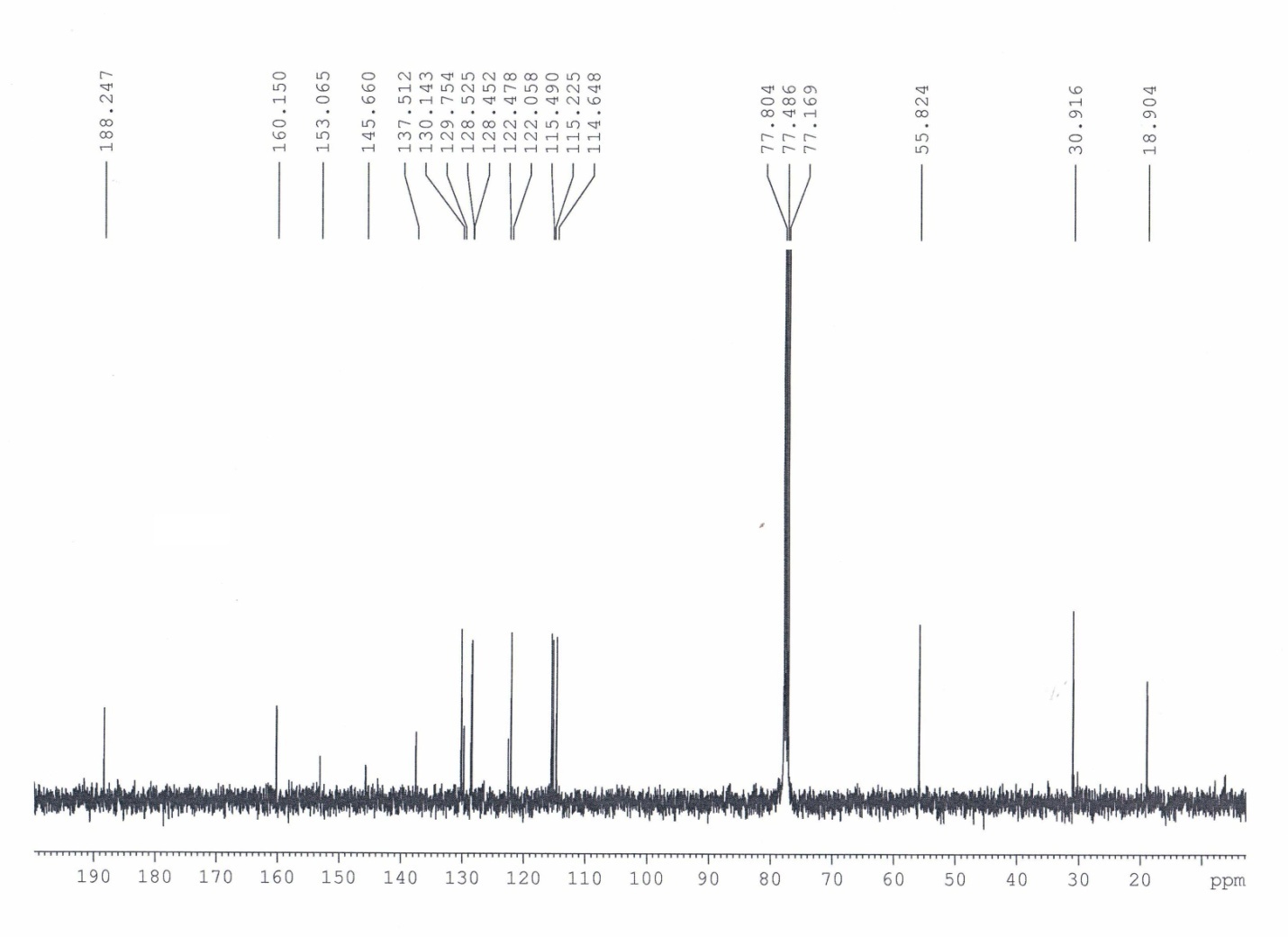


**3f**
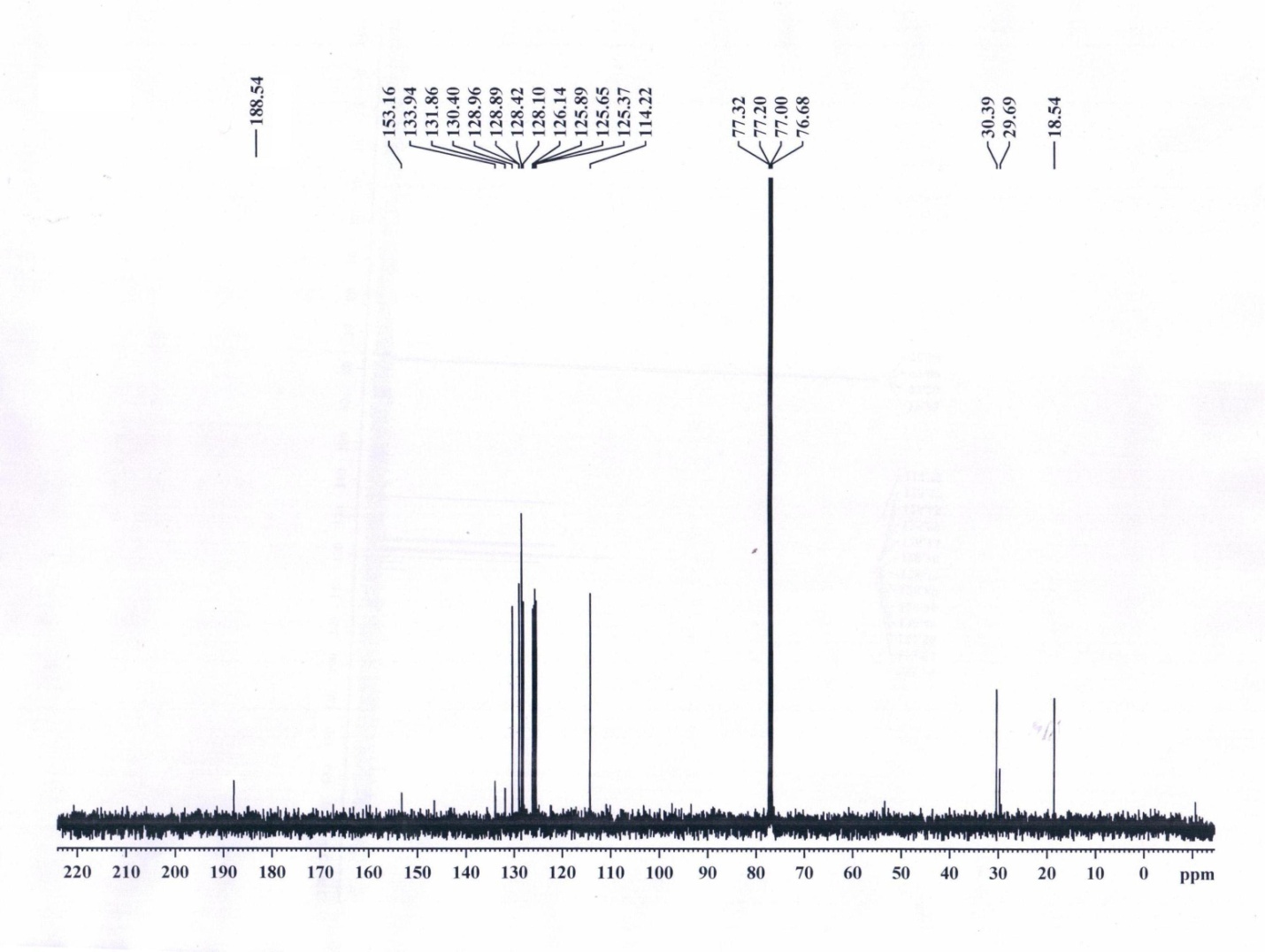


**3j**
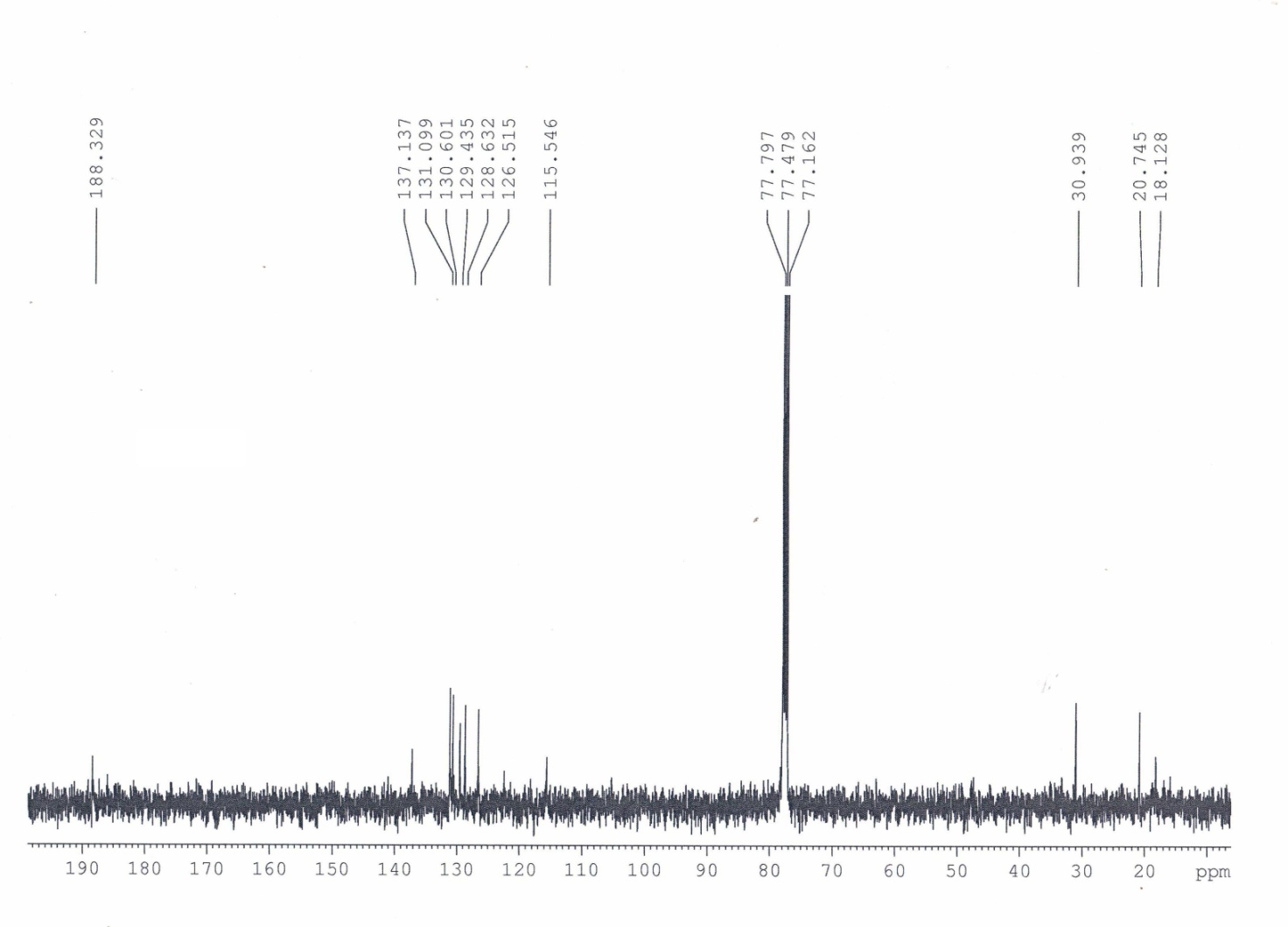


**3c LC/MS 250.3 (M+1)**


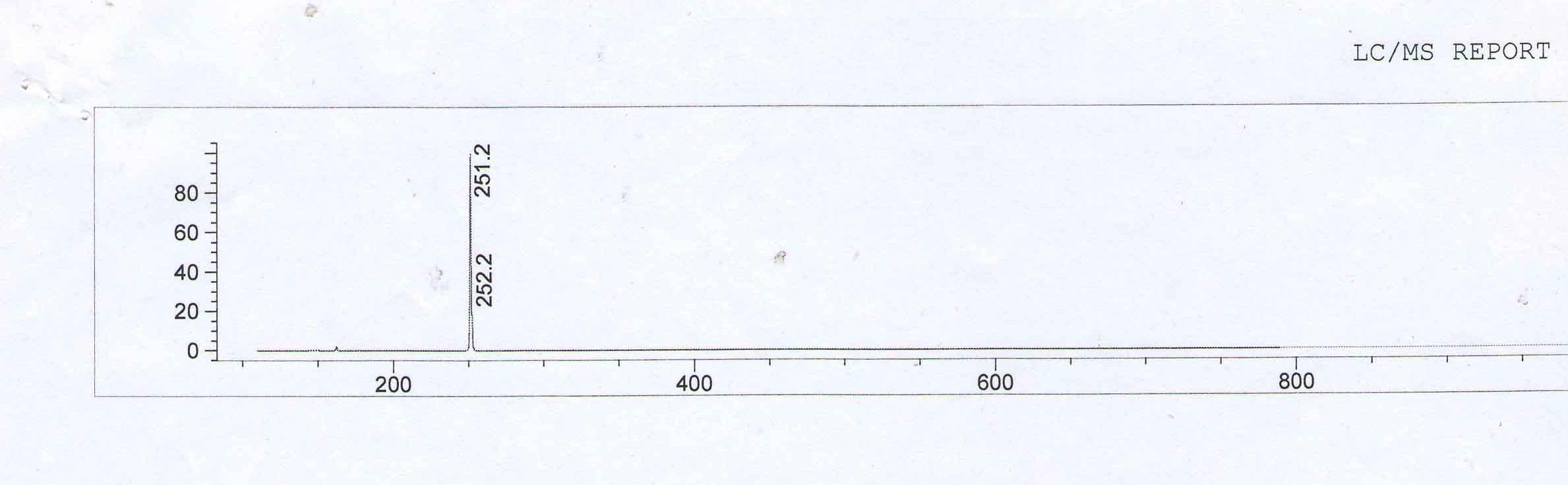


**3g LC/MS 284.72**
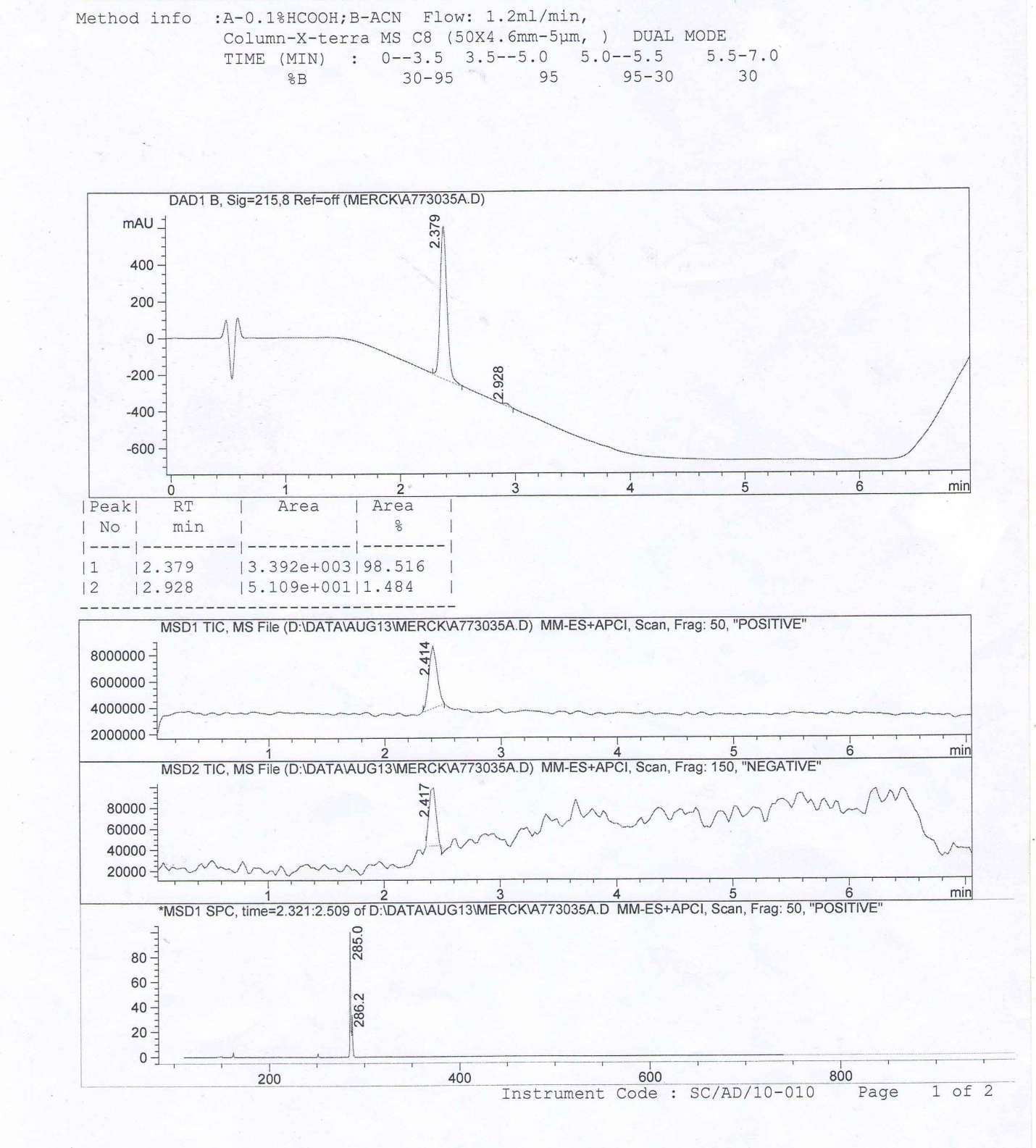


**3k LC/MS 318.1**


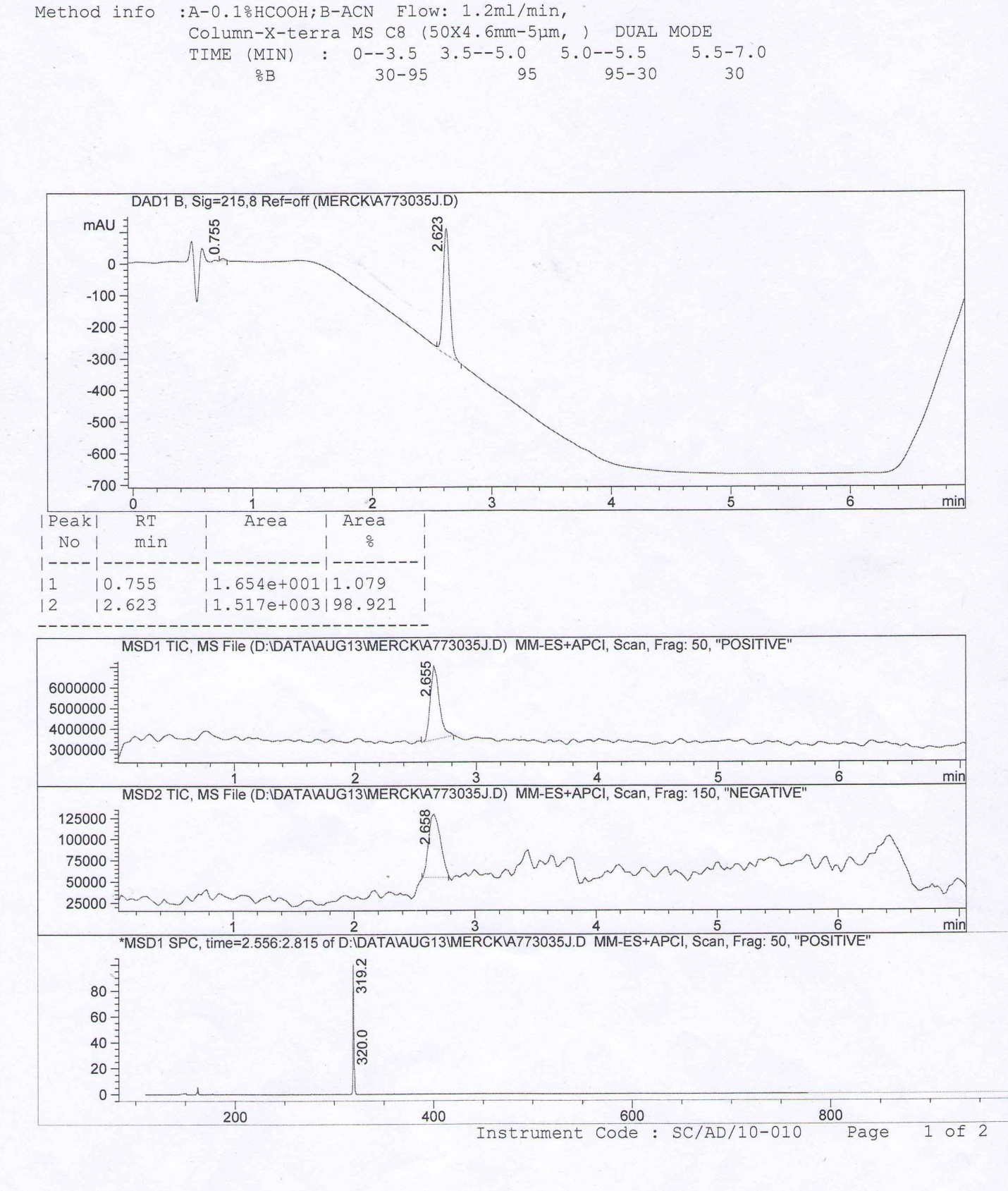

Supplement: S1 Data — (DOCX) [file pone.0131896.s001.docx]
