# Supplementary material for: A One Pot Synthesis of Novel Bioactive Tri-Substitute-Condensed-Imidazopyridines that Targets Snake Venom Phospholipase A2
Source: PLoS One. 2015 Jul 21;10(7):e0131896. doi: 10.1371/journal.pone.0131896 (PMC4511007; doi:10.1371/journal.pone.0131896)
Supplement: S2 Data — (DOCX) [file pone.0131896.s002.docx]

Data S2

Structural analysis of new molecules

2a: 1-(8-Bromo-2-methyl-imidazo[1,2-α]pyridine-3-yl)-ethanone:

Buff solid, mp=141-142^º^C. (81%) IR KBr, ν_max_/cm^-1^ 1632, 1581, ^1^H NMR (400 MHz, CDCl_3_): δ 9.78 (d, J=6.8, 1H), 7.77 (d, J=7.6, 1H) 6.97 (t, J=14.4, 1H), 2.92 (s, 3H), 2.67(s, 3H) ppm; LC-MS: [M+2] 256.5;

Anal.Calcd for C_10_H_9_ BrN_2_O: C, 47.46; H, 3.58; N, 11.07. Found: C, 47.18; H, 3.61; N, 10.56.

3a:1-[8-(4-Chloro-3-trifloromethyl-phenyl)-2-methyl-imidazo[1,2-α]pyridine-3yl]ethanone: White solid, mp= 141-143^º^C. (70%), IR KBr, ν_max_/cm^-1^ 1630, 1573, ^1^H NMR (400 MHz, CDCl_3_): 9.84 (d,1H,Ar-H), 8.24-8.22(d, 1H,Ar-H),8.13-8.11(m,1H,Ar-H), 7.67-7.65 (d,1H,Ar-H), 7.60-7.58 (d,1H,Ar-H),7.18-7.14 (t,1H,Ar-H), 2.86 (s, 3H,O=C-CH_3_), 2.66 (s, 3H,-CH_3_); ^13^C NMR(400 MHz, CDCl_3_); δ 188.40,161.15, 153.36, 135.16, 133.91, 132.85, 129.27,128.67,128.16, 127.20,115.01, 30.91, 18.89 ppm; LC-MS: [M+1] 353.65;

Anal.Calcd for C_17_H_12_ClF_3_N_2_O: C, 57.88; H, 3.43; N, 7.94. Found: C, 57.25; H, 3.63; N, 7.42.

3b: 1-[8-(4-Benzyloxy-3-fluoro-phenyl)-2methyl-imidazo[1,2-α]pyridine-3-yl]-ethanone.

Solid, mp=154-156^º^ C (76%). IR KBr, ν_max_/cm^-1^ 1636, 1591, 1H NMR (400 MHz, CDCl3): 9.87(d,1H,Ar-H), 7.69-7.67 (d,1H,Ar-H)), 7.64-7.21(m, 9H), 5.23 (s, 2H), 3.07 (s, 3H), 2.7 (s, 3H) ppm; LC-MS: [M+1] 375.80.

Anal.Calcd for C_23_H_19_FN_2_O_2_ : C, 73.78; H, 5.11; N, 7.48. Found: C, 73.11; H, 5.19; N, 7.12.

3c: 1-(2-Methyl-8-phenyl-imidazo[1,2-α]pyridine-3-yl)-ethanone.

Solid, mp=160-162^º^ C (75%). IR KBr, ν_max_/cm^-1^ 1626, 1572, ^1^H NMR (400 MHz, CDCl_3_) δ 9.84 (d, 1H,Ar-H), 7.87-7.19 (m, 7H,Ar-H), 2.93 (s, 3H,O=C-CH_3_), 2.66 (s, 3H,-CH_3_) ppm; LC-MS: [M+1] 251.2;

Anal.Calcd for C_16_H_14_ N_2_O: C, 76.78; H, 5.64; N, 11.19. Found: C, 76.09; H,5.82; N, 11.17.

3d: 1-[8-(3-chloro-phenyl)-2-methyl-imidazo[1,2-α]pyridine-3-yl]-ethanone.

Solid, mp=114-116^º^ C (80%). IR KBr, ν_max_/cm^-1^ 1609, 1484, ^1^H NMR (400 MHz, CDCl_3_) δ 9.80 (d,1H,Ar-H), 7.92-7.09 (m, 6H,Ar-H), 2.83 (s, 3H,O=C-CH_3_), 2.66 (s, 3H,-CH_3_)ppm; LC-MS: [M+1] 285.2;

Anal.Calcd for C_16_H_13_ ClN_2_O: C, 67.49; H, 4.60; N, 9.84. Found: C, 67.01; H, 4.20; N, 9.45.

3e. 1-[8-(3-Methoxy-phenyl)-2-methyl-imidazo[1,2-α]pyridine-3yl]-ethanone.

Solid, mp=116-119^º^ C (81%). IR KBr, ν_max_/cm^-1^ 1621, 1597, ^1^H NMR (400 MHz, CDCl_3_); δ 9.87(d,1H,Ar-H), 7.66-7.04(m, 6H,Ar-H), 3.93 (s, 3H,O-CH_3_), 2.96 (s, 3H,O=C-CH_3_), 2.71 (s, 3H,-CH_3_) ppm; ^13^C NMR( 400 MHz, CDCl_3_); δ 188.24, 160.15, 153.06, 145.66, 137.51, 130.14 129.75, 128.52, 128.45, 122.47, 122.05, 115.49, 115.22, 114.64, 55.82, 30.91, 18.90 ppm; LC-MS: [M+1] 281.20;

Anal.Calcd for C_17_H_16_N_2_O_2_ : C, 72.84; H, 5.75; N, 9.99. Found: C, 72.59; H, 5.88; N, 9.62.

3f. 1-(2-Methyl-8-naphthalen-1-yl-imidazo [1,2-α]pyridine-3-yl)-ethanone

Solid, mp=179-182º C (86%). IR KBr, ν_max_/cm^-1^ 1619, 1497, ^1^H NMR (400 MHZ, CDCl_3_); δ 10.0(d,1H,Ar-H), 8.07-7.40 (m, 9H,Ar-H), 3.01(s, 3H,O=C-CH_3_), 2.71(s, 3H,-CH_3_) ppm; ^13^C NMR( 400 MHz, CDCl_3_); δ 188.14,160.14, 153.16, 133.94, 131.86, 130.40, 128.92, 128.10, 126.14, 125.89, 125.65, 125.37, 114.22, 30.39, 18.54 ppm; LC-MS: [M+1] 301.49;

Anal.Calcd for C_20_H_16_ N_2_O: C, 79.98; H, 5.37; N, 9.33. Found: C, 79.41; H, 5.50; N, 8.98.

3g. 1-[8-(4-chloro-phenyl)-2-methyl-imidazo[1,2-α]pyridine-3-yl]-ethanone.

Solid, mp=111-114^º^c (82%). IR KBr, ν_max_/cm^-1^ 1623, 1565, ^1^H NMR (400 MHZ, CDCl_3_); δ 9.84(d, 1H,Ar-H), 7.87-7.18 (m, 6H,Ar-H), 2.90 (s, 3H,O=C-CH_3_), 2.66 (s, 3H,-CH_3_); LC-MS: [M+1] 286.2;

Anal.Calcd for C_16_H_13_ ClN_2_O: C, 67.49; H, 4.60; N, 9.84. Found: C, 67.38; H, 4.71; N, 9.49.

3h. 4-(3-Acetyl-2-methyl-imidazo[1,2-α]pyridine-8-yl)-N-cyclopentyl-2-ethyl-butyramide.

Solid, mp=117-119^0^c (87%).IR KBr, ν_max_/cm^-1^ 1630, 1582, 1493, ^1^H NMR (400 MHz,); δ 9.70 (d, J=7.2, 1H), 8.19(s, 1H, NH), 7.92-7.82(m, 2H), 4.26(m, 1H), 2.78-2.51(m, 2H), 2.62(s, 3H), 2.51(s, 3H), 1.92-1.55(m, 13H), 0.94(t, 3H) LC-MS: [M+1] 356.78;

Anal.Calcd for C_21_H_29_N_3_O_2_ : C, 70.95; H, 8.22; N, 11.82. Found: C, 70.20; H, 8.01; N, 11.31.

3i. 1-[8-(2-Fluoro-3-methoxy-phenyl)-2-methyl-imidazo[1,2-α]pyridine-3-yl]-ethanone

Solid, mp=190-192˚ C (86%). IR KBr, ν_max_/cm^-1^ 1636, 1588, ^1^H NMR (400 MHz, CDCl_3_); δ 9.82(d,1H,Ar-H), 7.56(d,1H,Ar-H), 7.27-7.06(m, 4H,Ar-H), 3.93(s, 3H,-OCH_3_), 2.82(s, 3H,O=C-CH_3_), 2.64(s, 3H,-CH_3_); LC-MS: [M+1] 299.2;

Anal.Calcd for C_17_H_15_FN_2_O_2_: C, 68.45; H, 5.07; N, 9.39. Found: C, 67.30; H, 5.17; N, 9.01.

3j. 1-(2-Methyl-8-o-tolyl-imidazo[1,2-α]pyridine-3-yl)-ethanone.

Solid, mp=157-159˚C (80%). IR KBr, ν_max_/cm^-1^ 1630, 1583, ^1^H NMR (400 MHz, CDCl_3_); δ 9.92(d,1H,Ar-H), 8.34(d,1H,Ar-H), 7.79-7.26(m, 5H,Ar-H), 3.01(s, 3H O=C-CH_3_), 2.69(s, 3H, ,-CH_3_), 2.19(s, 3H,-CH_3_)ppm; ^13^C NMR( MHz, CDCl_3_); δ 188.32,160.54, 155.74, 144.68, 137.13,131.09,130.60, 129.43, 128.63, 126.51, 115.54, 30.93, 20.74, 18.12.

LC-MS: [M+1] 265.56; Anal.Calcd for C_17_H_16_N_2_O: C, 77.25; H, 6.12; N, 10.60. Found: C, 76.95; H,5.98; N, 10.55.

3k. 1-[2-Methyl-8-(4-trifluoromethyl-phenyl)-imidazo[1,2-α]pyridine-3-yl]-ethanone

Solid, mp=121-123˚C (76%). IR KBr, ν_max_/cm^-1^ 1638, 1581, ^1^H NMR (400 MHz, CDCl_3_); δ 9.86(d,1H,Ar-H), 8.04-8.02(dd,2H,Ar-H),7.79-7.77(dd,2H,Ar-H),7.63-7.61(d,1H,Ar-H),7.21-7.17(m,1H,Ar-H),2.89(s,3H,O=C-CH_3_),2.67(s,3H,-CH_3_)ppm; LC-MS: [M+1] 319.2;

Anal.Calcd for C_17_H_13_N_2_O: C, 64.15; H, 4.12; N, 8.80. Found: C, 64.11; H, 3.84; N, 8.38

3l. 1-[8-(4-Ethyl-phenyl)-2-methyl-imidazo[1,2-α]pyridine-3-yl]ethanone.

Solid, mp=169-172˚C (79%). IR KBr, ν_max_/cm^-1^ 1636, 1584, ^1^H NMR (400 MHz, CDCl_3_); δ 9.83(d,1H), 7.78-7.77(dd, 2H,Ar-H),7.66-7.64(d,1H,Ar-H),7.39-7.37(dd,2H,Ar-H),7.26-7.22(m,1H,Ar-H), 2.99(s,3H,O=C-CH_3_), 2.76-2.70(m,2H,-CH_2_),2.67(s,1H,-CH_3_) 1.28(t,m,3H,-CH_3_)ppm; LC-MS: [M+1] 279.2

Anal.Calcd for C_18_H_18_N_2_O: C, 77.67; H, 6.52; N, 10.06, Found: C, 76.17; H, 6.15; N, 10.01.
